# The worldwide trend in diabetes awareness, treatment, and control from 1985 to 2022: a systematic review and meta-analysis of 233 population-representative studies

**DOI:** 10.3389/fpubh.2024.1305304

**Published:** 2024-05-17

**Authors:** Ehsan Shahrestanaki, Nami Mohammadian Khonsari, Ehsan Seif, Fereshteh Baygi, Hanieh-Sadat Ejtahed, Ali Sheidaei, Shirin Djalalinia, Dianna J. Magliano, Mostafa Qorbani

**Affiliations:** ^1^Non-communicable Diseases Research Center, Alborz University of Medical Sciences, Karaj, Iran; ^2^Department of Epidemiology, School of Public Health, Iran University of Medical Sciences, Tehran, Iran; ^3^Research Unit of General Practice, Department of Public Health, University of Southern Denmark, Odense, Denmark; ^4^Obesity and Eating Habits Research Center, Endocrinology and Metabolism Clinical Sciences Institute, Tehran University of Medical Sciences, Tehran, Iran; ^5^Endocrinology and Metabolism Research Center, Endocrinology and Metabolism Clinical Sciences Institute, Tehran University of Medical Sciences, Tehran, Iran; ^6^Department of Epidemiology and Biostatistics, Tehran University of Medical Sciences, Tehran, Iran; ^7^Deputy of Research & Technology, Ministry of Health & Medical Education, Tehran, Iran; ^8^School of Public Health and Preventive Medicine, Monash University, Melbourne, VIC, Australia; ^9^Baker Heart and Diabetes Institute, Melbourne, VIC, Australia; ^10^Chronic Diseases Research Center, Endocrinology and Metabolism Population Sciences Institute, Tehran University of Medical Sciences, Tehran, Iran

**Keywords:** diabetes, awareness, treatment, control, systematic review, meta-analysis

## Abstract

**Background:**

With the rapid increase in the prevalence of DM, studies on the awareness, treatment, and control of this condition are essential. Therefore, this study aimed to review the literature and pool the awareness, treatment, and control of diabetes at the global, regional, and national levels.

**Methods:**

In this systematic review and meta-analysis, several databases, including MEDLINE/PubMed, Institute of Scientific Information (ISI), Scopus, and Google Scholar, were searched using appropriate keywords up to June 2022. Observational studies investigating the awareness, treatment, and control of glucose levels among diabetic individuals were included. Awareness, treatment, and control were defined as the proportion of participants who were aware of their diabetes condition, treated pharmacologically, and achieved adequate glucose control, respectively. Two investigators independently conducted the study selection, data extraction, and quality assessment. Heterogeneity among studies was calculated using Chi-square, and a random-effect meta-analysis was used to pool the rates.

**Results:**

A total of 233 studies published between 1985 and 2022 met the inclusion criteria. The included studies had a combined population of 12,537,968. The pooled awareness of DM was 60% (95%CI: 56–63) and ranged from 41% (25–57) in low-income countries to 68% (64–72) in high-income countries, with no significant trend observed over the assessed periods at the global level. The pooled treatment of DM globally was 45% (42–48) and varied from 37% (31–43) in lower-middle-income countries to 53% (47–59) in high-income countries, showing variation over the examined time period. Before 2000, the proportion of adequate DM control was 16% (12–20), which significantly improved and reached 22% (19–25) after 2010. The pooled awareness, treatment, and control of DM were higher in females, high-income countries, and urban areas compared to males, upper and lower-middle-income countries, and rural areas, respectively. The older adults population had higher awareness and treatment rates than the adult population, but their DM control did not differ significantly.

**Conclusion:**

Despite the high level of awareness and treatment among the diabetic population, treatment success (control) is considerably low, particularly in low-income countries and rural areas. It is crucial to improve awareness, treatment, and control by strengthening the primary care system in all countries.

## Introduction

Type 2 diabetes mellitus (DM) is a metabolic disorder and a major risk factor for many other diseases, resulting in both long-term and short-term complications. Diabetes, along with its associated complications, can lead to mortality and severe morbidity (1). In 1980, the number of people aged 18–99 years with diabetes was 108 million, which increased to 451 million in 2017. Unfortunately, these numbers are projected to increase to 693 million by 2045. The prevalence of diabetes has been rapidly increasing, especially in low- and middle-income countries. Additionally, the burden, treatment, and costs of DM and its complications are increasing at a fast pace (2).

Education on the early signs of diabetes, along with effective screening programs, is crucial in identifying DM in its early stages. This results in increased awareness, timely treatment, and a significant reduction in diabetes complications (3). The treatment options for diabetes vary based on several factors, including the severity and duration of DM, individual circumstances, and the level of social disadvantage. These options range from inexpensive dietary and lifestyle changes in the early stages to the use of expensive multiple glucose-lowering medications and insulin treatment in the later stages of the disorder (4–6). Therefore, if managed appropriately, diabetes could lead to significantly fewer complications, premature deaths, and less burden on the government and healthcare system (7).

With the rapid increase in the prevalence of DM, studies on the awareness, treatment, and control of this condition are essential to illuminate a path for governments and public health officials worldwide to manage this illness more effectively. There have been no systematic reviews and meta-analyses based on individual patient data (IPD) or aggregated data on a global scale over the past few years. Thus, this study aimed to examine the long-term awareness, treatment, and control of DM in the general population at the national, regional, and global levels.

## Material and methods

This systematic review and meta-analysis was conducted following the guidelines outlined in the Cochrane Handbook for Systematic Reviews of Interventions (8). Furthermore, the study’s findings were reported in accordance with the Preferred Reporting Items for Systematic Review and Meta-Analysis (PRISMA) statement (9).

### Search strategy

Several international databases, including MEDLINE/PubMed, the Institute of Scientific Information (ISI), Scopus, and Google Scholar, were searched up until June 2022. The search strategy was developed based on the outcomes (awareness, treatment, and control of diabetes). The databases were searched using keywords such as “diabetes,” “awareness,” “treatment,” and “control,” along with related keywords based on each database’s search strategy. The search strategy in the selected databases is presented in Supplementary Appendix Table 1. Additionally, the reference lists of the included articles were manually searched. The search results from all databases were imported into EndNote software, and duplicate studies were removed. The search was conducted by one researcher and approved by a second researcher, following the search strategy.

### Eligibility criteria

All cross-sectional studies that investigated the awareness, treatment, and control of glucose levels among individuals with diabetes were included. Studies with the following characteristics were excluded: (1) participants below 18 years old, (2) type 1 diabetes, (3) non-English studies, and 4) case reports, case series, and letters to the editor studies. If a study did not specify the type of study, it was still included.

### Definition of outcomes

DM was defined as having fasting blood sugar (FBS) above the cutoff level specified by the studies, a self-reported previous diagnosis of DM by a healthcare professional, or being on pharmacological treatment for DM. Awareness was defined as the proportion of participants who were aware of their condition with DM. Treatment was defined as the proportion of participants who were treated with medication due to having DM (adherence to treatment). Control was defined as the proportion of participants whose DM was adequately controlled. Control was calculated using two types of denominators: control among all diabetic individuals or DM control among treated diabetic individuals.

### Data extraction

Two researchers independently extracted the data using an electronic data extraction form. The following characteristics and outcomes of the included studies were extracted: first author, year of publication, country, study design, living area (urban or rural), sample size, gender, age (mean ± standard deviation (SD) or range), definition of diabetes, awareness, treatment, and DM control, along with their 95% confidence interval (CI). Any disagreements between the two researchers were resolved through discussion or with the involvement of a third researcher.

### Quality assessment (Q.A)

The Newcastle–Ottawa scale for cross-sectional studies was used to assess the quality of the included studies. This scale evaluates eight items in three main domains: selection, comparability, and outcome. The Q.A score, which is the sum of the scores of each item, ranges from 0 to 10. Higher scores indicate a lower risk of bias. On this scale, low-quality studies with a high risk of bias were assigned scores ranging from 0 to 3, middle-quality studies with a medium risk of bias were assigned scores from 4 to 6, and high-quality studies with a low risk of bias were assigned scores from 7 to 10. Any disagreements between the two researchers regarding the Q.A score were resolved through discussion or with the involvement of a third researcher.

### Statistical analysis

The estimates were pooled using the “Metaprop” command in Stata. Heterogeneity among the studies was assessed using chi-square-based Q-tests. If heterogeneity was statistically significant, a random-effects model was used; otherwise, a fixed-effects model was used. Subgroup analyses were conducted based on gender, study population (adult/older adults), country income (classified according to the World Bank’s Classification) (10), living area (urban or rural), and Q.A score. All analyses were stratified by time periods (before 2000, 2001–2010, and after 2010). Meta-regression was performed to identify the source of heterogeneity among studies. Stata 17 (Copyright Stata Corp., LP, United States) was used for the meta-analysis. All figures were generated using R version 4.1.3 statistical software.

### Role of the funding source

The funding body had no involvement in the preparation, investigation, design, interpretation of the findings, or drafting of the study.

## Results

**Figure 1:**
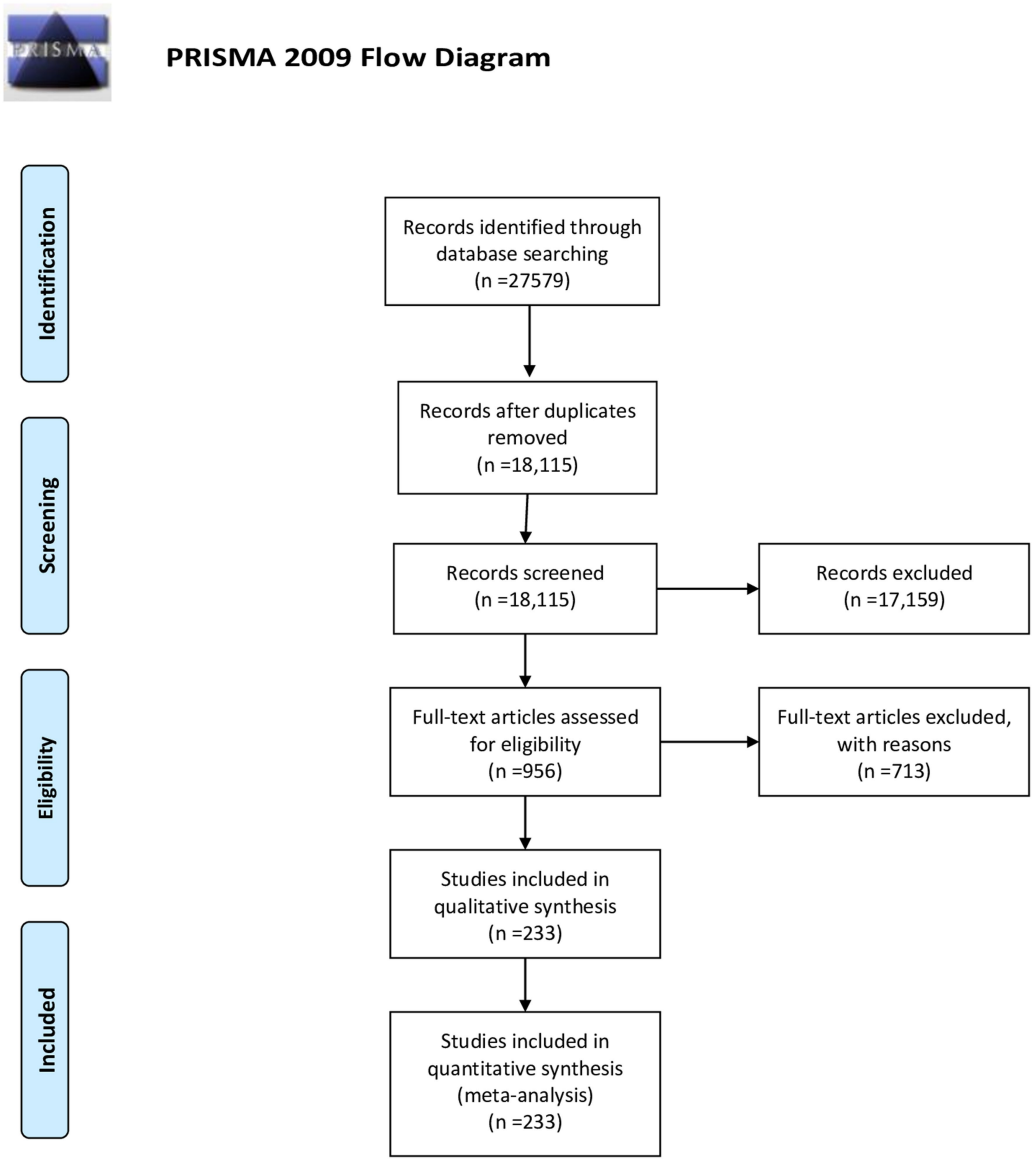
Study selection process based on PRISMA chart.

A total of 27,579 documents were retrieved from the search across all databases. After removing duplicate and irrelevant studies based on titles, abstracts, and full texts, 953 studies were reviewed for eligibility criteria. Finally, 233 studies met the inclusion criteria for qualitative and quantitative analyses (11–242). The study selection process is illustrated in Figure 1.

### Qualitative synthesis

The characteristics of the included studies and the reported awareness, treatment, and control of DM are presented in Supplementary Appendix Table 2. These studies were published between 1985 and 2022 and encompassed 84 countries and 2 regions (the Southern Cone of Latin America and South Asia). The majority of studies were conducted in China (34 studies), the United States (20 studies), Iran (15 studies), and India (13 studies). The combined total population of all included studies was 12,537,968, with a mean age of 48.7 (SD 10) among the participants. The highest reported levels of awareness, treatment, and control were observed in China (92%), the United States of America (87%), and China (76%), respectively.

### Quantitative synthesis

#### Heterogeneity

A significant level of heterogeneity was observed among the included studies regarding awareness, treatment, and control of DM (*p*-value <0.001). Therefore, the random-effects model was used to pool the proportions.

### Global level awareness, treatment, and control of DM

**Figure 2:**
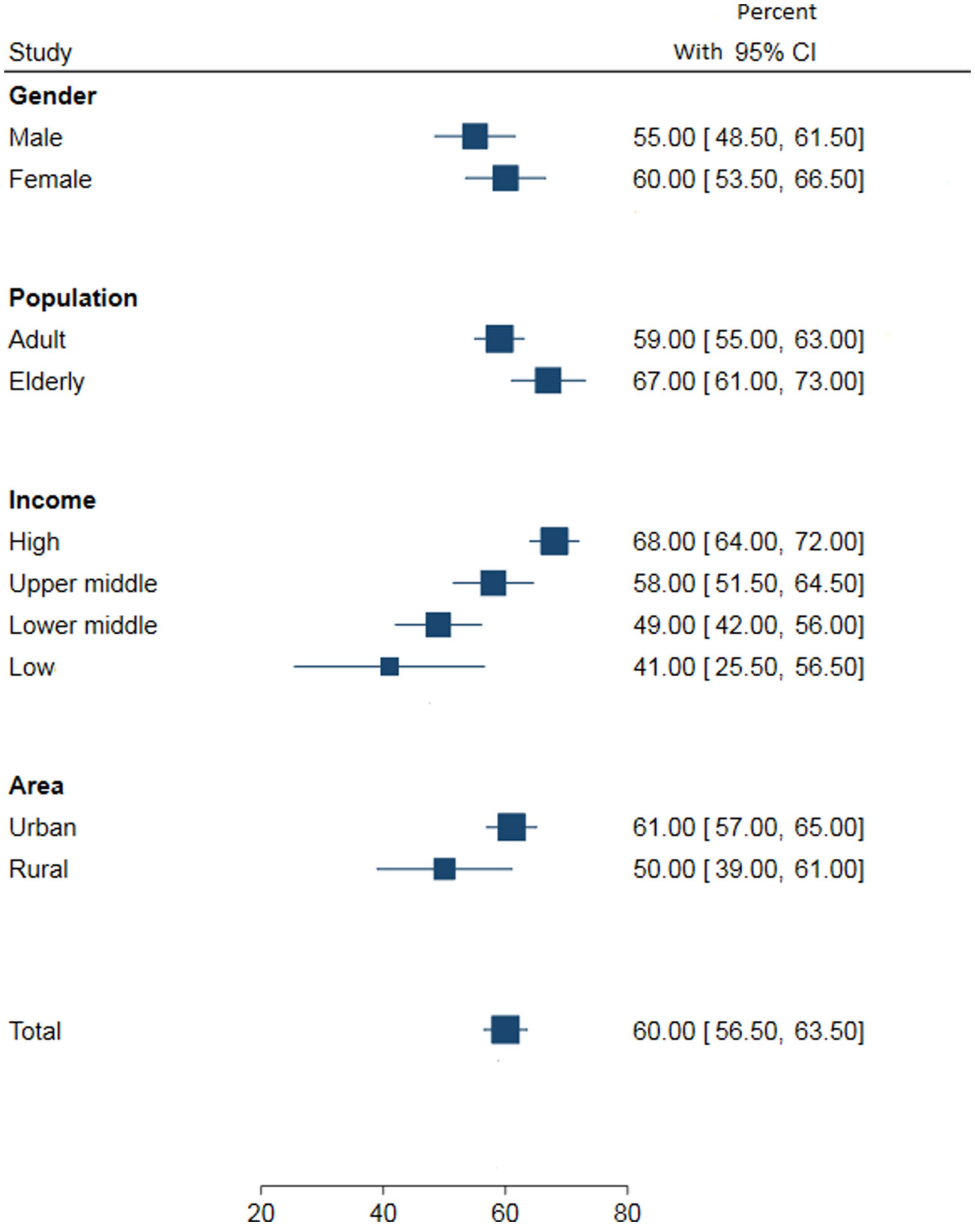
The pooled DM awareness by gender, study population, country’s income and living area at the global level.

**Figure 3:**
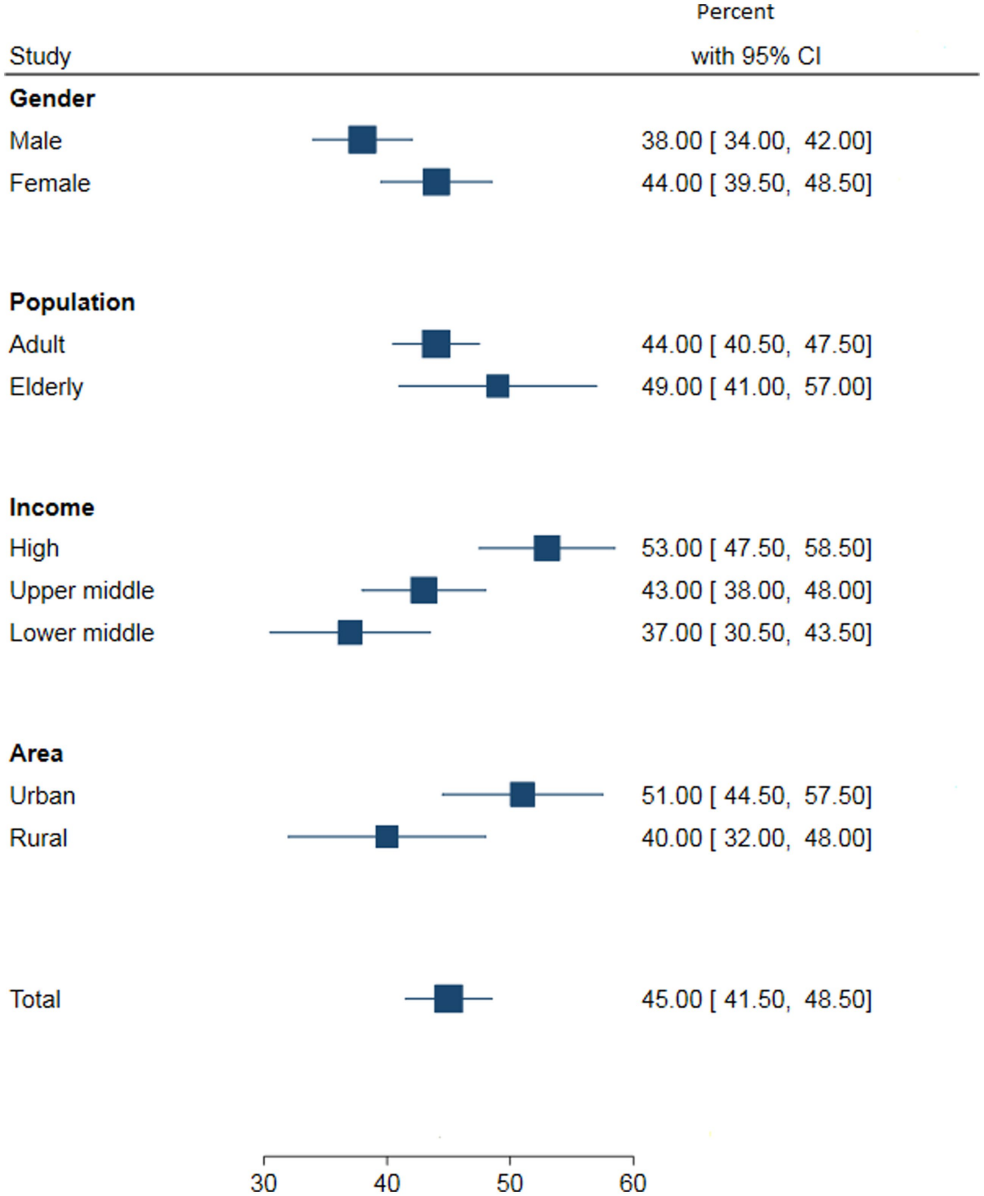
The pooled DM treatment by gender, study population, country’s income and living area at the global level.

**Figure 4:**
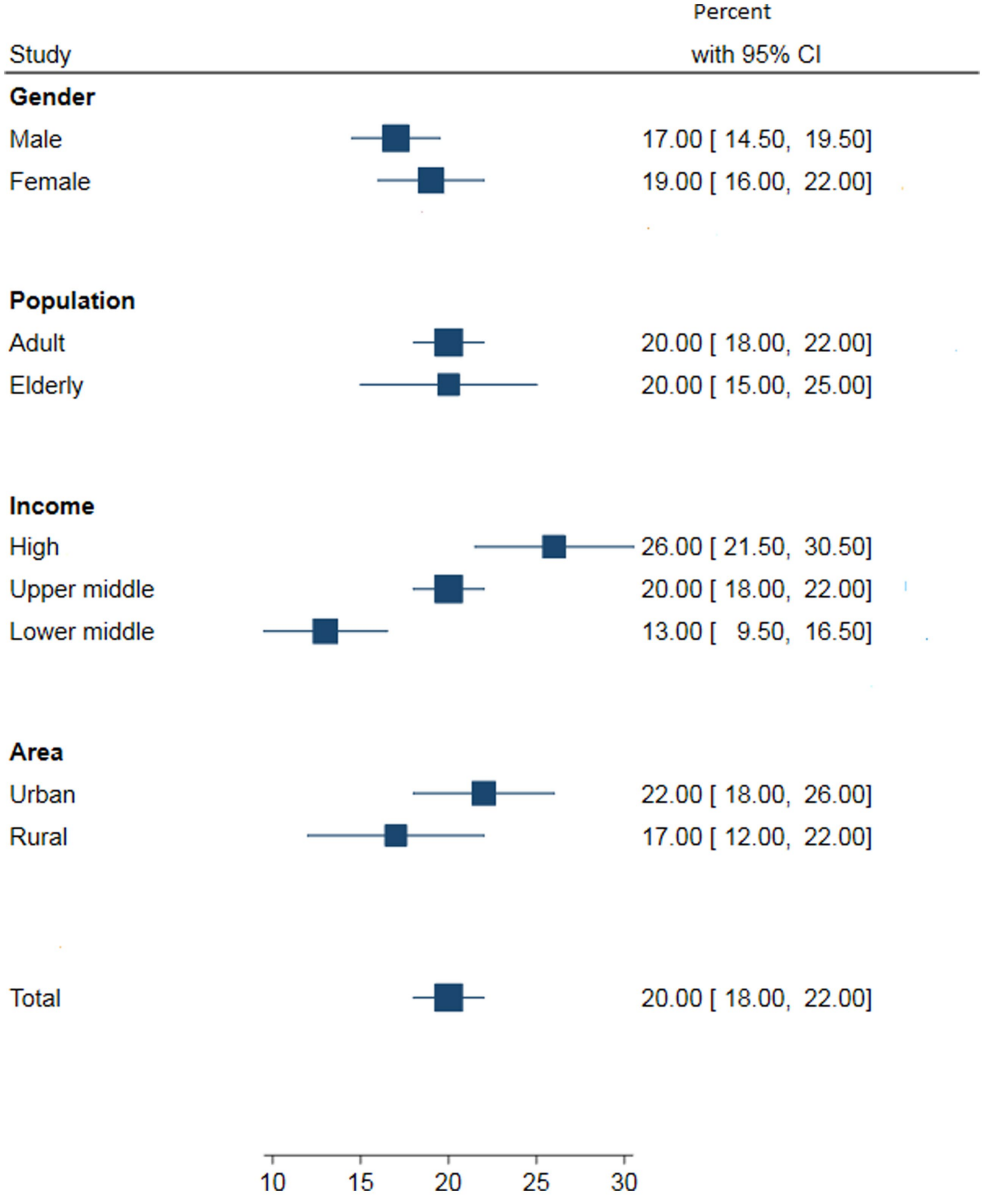
The pooled DM control by gender, study population, country’s income and living area among all the diabetic individuals at the global level.

**Figure 5:**
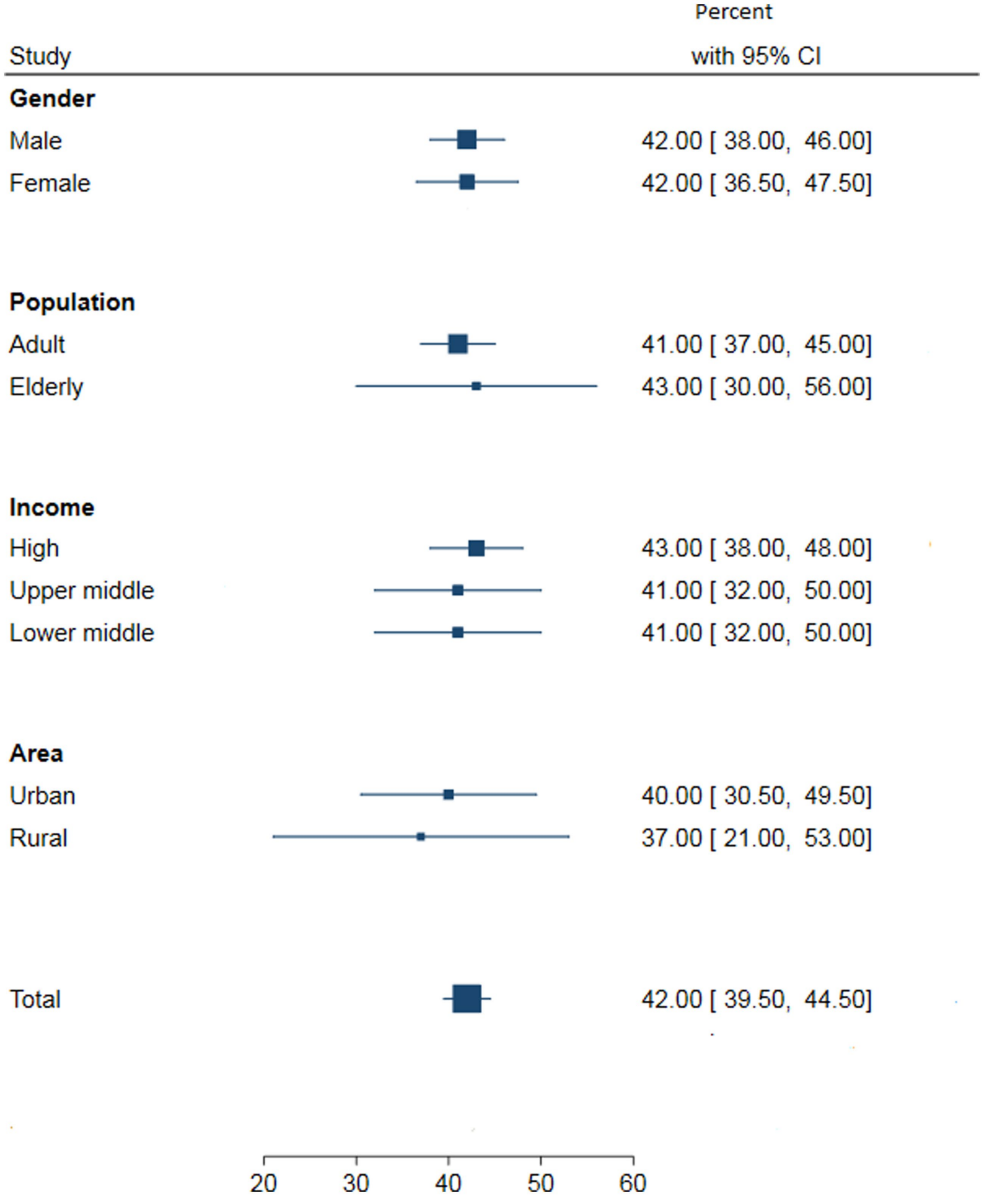
The pooled DM control by gender, study population, country’s income and living area among those receiving treatment at the global level.

Figures 2–5 present the pooled awareness, treatment, and control rates stratified by gender, study population, living area, and country’s income. The pooled awareness and treatment rates at the global level were 60% (95% CI: 56–63) and 45% (95% CI: 42–48), respectively. The proportion of DM control among all diabetic individuals was 20% (95% CI: 18–22), while among those receiving treatment, it was 42% (95% CI: 39–44).

### Awareness, treatment, and control of DM by the time period

Table 1 displays the meta-analysis results for the pooled estimates of awareness, treatment, and control of DM according to three study periods. Although the awareness of diabetes showed a decreasing trend across the three study periods, it was not statistically significant (before 2000: 61% (95% CI: 56, 66); 2000 to 2010: 60% (95% CI: 53, 66); after 2010: 59% (95% CI: 54, 64)). In all three periods, female subjects had higher awareness compared to male subjects. Awareness also increased with income level and was higher in urban and older adults populations compared to rural and adult populations, respectively.

**Table 1:** The proportion of awareness, treatment and control of diabetes according to gender, quality assessment, population, country incomes and study area.

	**Before 2000 year**				**2001–2010 year**				**After 2010 year**			
	**Pooled proportion (95% CI)**				**Pooled proportion (95% CI)**				**Pooled proportion (95% CI)**			
	**Awareness**	**Treatment**	**Control**^**1**^	**Control**^**2**^	**Awareness**	**Treatment**	**Control**^**1**^	**Control**^**2**^	**Awareness**	**Treatment**	**Control**^**1**^	**Control**^**2**^
Overall	61 (56, 66)	47 (39, 55)	16 (12, 20)	32 (26, 39)	60 (53, 66)	41 (37, 45)	19 (16, 23)	44 (41, 47)	59 (54, 64)	47 (42, 52)	22 (19, 25)	44 (40, 48)
**Gender**												
Male	59 (51, 67)	51 (31, 71)	21 (14, 28)	41 (36, 46)	55 (44, 66)	35 (25, 45)	16 (11, 21)	44 (39, 50)	53 (47, 59)	38 (34, 43)	16 (13, 20)	41 (36, 46)
Female	63 (53, 73)	56 (30, 81)	22 (12, 33)	41 (34, 48)	60 (48, 72)	40 (29, 50)	19 (13, 24)	44 (39, 49)	58 (52, 64)	45 (40, 49)	19 (15, 23)	40 (33, 48)
**Quality assessment**												
High	59 (50, 68)	39 (22, 56)	9 (1, 17)	17 (4, 29)	66 (59, 72)	33 (28, 38)	16 (12, 21)	40 (33, 46)	62 (51, 72)	40 (31, 49)	19 (13, 24)	41 (35, 46)
Moderate	62 (54, 71)	51 (39, 64)	18 (12, 24)	36 (27, 45)	59 (49, 69)	45 (37, 52)	20 (16, 25)	45 (42, 48)	58 (53, 62)	49 (43, 55)	21 (18, 24)	43 (37, 49)
Low	56 (39, 73)	_	_	_	28 (8, 64)	_	_	_	64 (47, 81)	57 (40, 74)	41 (18, 64)	64 (40, 87)
**Study population**												
Adult	60 (55, 66)	48 (39, 57)	15 (11, 19)	31 (24, 37)	60 (53, 67)	40 (36, 45)	19 (16, 23)	44 (41, 47)	58 (53, 63)	47 (42, 51)	22 (19, 25)	45 (40, 49)
older adults	66 (57, 74)	46 (43, 49)	_	_	60 (49, 71)	50 (38, 61)	20 (14, 26)	45 (29, 61)	75 (70, 80)	53 (20, 86)	19 (1, 37)	34 (16, 52)
**Country’s incomes**												
High	68 (64, 71)	53 (41, 64)	17 (11, 24)	31 (21, 41)	67 (63, 71)	45 (38, 52)	26 (19, 33)	49 (43, 56)	71 (58, 84)	65 (60, 70)	36 (28, 44)	55 (37, 72)
Upper middle	49 (37, 61)	33 (16, 50)	11 (7, 16)	36 (23, 49)	56 (46, 66)	40 (32, 48)	17 (14, 21)	41 (37, 45)	62 (57, 68)	48 (41, 54)	22 (19, 26)	45 (40, 49)
Lower middle	49 (38, 60)	_	_	_	49 (35, 63)	38 (25, 52)	17 (9, 17)	44 (36, 51)	49 (41, 56)	34 (29, 39)	11 (8, 13)	34 (29, 39)
Low income	_	_	_	_	41 (11, 72)	_	_	_	41 (23, 60)	_	_	_
**Living area**												
Urban	59 (48, 69)	46 (33, 60)	14 (9, 19)	27 (24, 29)	68 (62, 73)	56 (47, 66)	24 (17, 31)	43 (37, 39)	58 (51, 66)	50 (37, 63)	22 (16, 27)	43 (38, 48)
Rural	41 (26, 56)	_	_	_	48 (23, 74)	41 (20, 62)	19 (9, 29)	41 (34, 49)	52 (42, 63)	41 (32, 50)	17 (10, 23)	36 (30, 42)

^1^Control among diabetes individuals. ^2^Control among treated diabetes individuals.

Regarding treatment, the pooled proportions were equal in the first and third study periods (47%). However, treatment rates increased from the second to the third period (41 to 47%). More women received treatment for diabetes than men in all three periods. Treatment rates were higher in high-income countries compared to upper- and lower-middle-income countries based on the country’s income. Additionally, treatment rates were higher in urban and older adults populations compared to rural and adult populations, respectively.

Among all diabetic individuals, the proportion of people with adequate DM control increased across the three periods (before 2000: 16%; 2000 to 2010: 19%; after 2010: 22%). The proportion of women with adequate DM control was higher than that of men. Based on the country’s income, high-income countries had significantly higher proportions of adequate DM control compared to upper- and lower-middle-income countries in all three periods. In terms of living area, urban populations had higher proportions of adequate DM control compared to rural populations. The reported proportions of adequate DM control were somewhat higher in the adult population compared to the older adults population in the third period, but almost the same in the second period.

The proportion of adequate DM control among treated diabetic individuals increased across the three periods (before 2000: 32%; 2000 to 2010: 44%; after 2010: 44%). There were no significant differences in diabetes management between men and women who were treated. The proportion of adequate DM control among those receiving treatment was significantly higher in high-income countries compared to upper- and lower-middle-income countries in the second and third periods. However, in the first period, this proportion was higher in upper-middle-income countries compared to high- and lower-middle-income countries. Urban populations had higher proportions of adequate DM control among treated individuals compared to rural populations. The reported proportion of adequate DM control among treated individuals was somewhat higher in the adult population compared to the older adults population in the third period, but almost the same in the second period.

**Figure 6:**
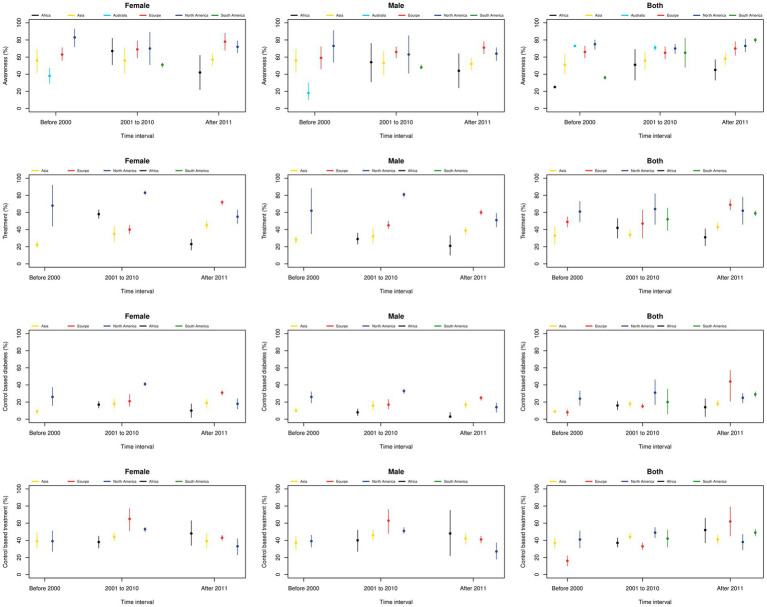
DM awareness, treatment, and control according to gender and time period at the regional level.

The awareness, treatment, and control of DM at the regional level (Figure 6) illustrates the regional-level awareness, treatment, and control of DM according to sex and time period. The highest awareness rates in male and female subjects were observed in the North American region, with proportions of 83 and 73% in the first and second studied periods, respectively. From 2001 to 2010, the highest awareness in male subjects was reported in Europe (66%), while in female subjects, it was estimated in North America (70%). After 2010, the highest awareness rates in male and female subjects were observed in Europe, with 71 and 78%, respectively.

Regarding treatment, the highest rates in both genders in the first and second studied periods were observed in North America (61 and 64%, respectively) and in Europe (69%) after 2010. By sex, the highest treatment rates in women were seen in North America (68, 83, and 55% in the three time periods, respectively), and in men in North America (62, 81%) in the first and second periods, and in Europe (60%) in the third period.

The highest proportion of adequate DM control among all diabetic individuals in the first and second periods was seen in North America, with proportions of 24 and 31%, respectively, and in Europe, with 44% in the third period. Moreover, the sex-stratified meta-analysis shows that the highest proportion of adequate DM control among women was seen in North America in the first (26%) and second (41%) periods, and in Europe (31%) in the third period. Among those using glucose-lowering treatments, both genders showed that North America had the highest control rates in the first and second studied periods, with proportions of 41 and 49%, respectively. However, after 2010, Europe had the highest proportion of adequate DM control (62%). The highest proportion of adequate DM control among women treated for diabetes was seen in Asia, followed by Europe and Africa during the three studied periods, respectively. For men, the highest proportion of adequate DM control was observed in North America, followed by Europe and then Africa in the third studied period.

### The awareness, treatment, and control of DM at the national level

The highest awareness rates among women were seen in the United States (83%), followed by Turkey (91%), and Mauritania (87%) during the three studied periods (Supplementary Appendix Figure 1). The highest awareness rates in men were observed in England (78%), South Africa (84%), and Mauritania (91%), during the three studied periods (Supplementary Appendix Figure 2).

The highest treatment rates in women were observed in the United States in the first and second periods, with proportions of 68 and 83%, respectively, and in France with a proportion of 82% in the third period (Supplementary Appendix Figure 3). In men, the highest treatment rates were seen in the United States in the first and second studied periods, with proportions of 62 and 81%, respectively, and in Nepal with a proportion of 65% in the third period (Supplementary Appendix Figure 4).

The highest proportion of adequate DM control among all diabetic women was seen in the United States, with proportions of 26 and 41% in the first two studied periods, respectively, and in Iran with a 31% proportion in the third decade (Supplementary Appendix Figure 5). In men, the highest proportion of adequate DM control was reported in the United States in the first and second studied periods (26 and 33%, respectively), and in Iran in the last period (25%) (Supplementary Appendix Figure 6).

The highest proportion of adequate DM control among treated women was seen in Guinea (43%), Italy (65%), and Angola (83%) during the three studied periods (Supplementary Appendix Figure 7). For men, the highest proportion was observed in the United States (39%), Italy (63%), and Cameroon (67%) during the three studied periods (Supplementary Appendix Figure 8).

## Discussion

To the best of our knowledge, this systematic review and meta-analysis is the first comprehensive study to estimate the global proportion of people with DM who are aware of their condition, receiving treatment, and achieving adequate control of their glucose levels. The pooled proportion of people with diabetes who are aware of their disease was 60%, varying from 41% in low-income countries to 68% in high-income countries. The proportion of people with diabetes who were on treatment was 45%, ranging from 37 to 53% in different countries. Besides, we observed that diabetes management was adequate only in 16% of patients before 2000 and 22% after 2010. It should be noted that we observed a high variation in the reported proportions among different countries (85, 145, 196). This variation could be attributed to methodological differences, as well as the variety of age, characteristics, lifestyle, and economic status of the participants, and the screening practices of the healthcare systems.

Despite the progress in the accuracy of diagnostic methods, the trend in the proportion of people who were aware of their diabetes was surprisingly low, even in developed countries. Assessing diabetes awareness in the Latin America region showed that only 50% of patients were aware of their diseases. Considering that the prevalence of diabetes continues to increase across these countries, more comprehensive diabetes assessments in national surveys have been suggested (243). Moreover, a meta-analysis regarding diabetes trends in China from 1979 to 2012 showed that there was no obvious improvement in awareness of diabetes despite an increase observed in diabetes prevalence (244) This highlights that extra efforts should be made to increase screening for diabetes in communities. This finding was concordant with previous studies, which show a trend in awareness of other NCDs such as hypertension over the past three decades (245, 246).

Regarding the trend in treatment proportions, the pooled proportions illustrating the ratio of diabetic individuals under treatment did not differ significantly from the first studied period to the last. It should be noted that during these years, the diagnostic thresholds for diabetes diagnosis changed, leading to a decrease in the proportion of people with diabetes who were treated. Additionally, improved screening methods and public education efforts resulted in a larger proportion of previously undiagnosed individuals being recognized. Despite this increase in the number of individuals with diabetes, treatment proportions did not differ significantly, suggesting that treatment rates have improved across these three periods.

The difference between diabetes awareness and treatment emphasizes that converting knowledge to change in behavior and performance is difficult and time-consuming. According to the transtheoretical model, changing a behavior is a process with five stages: precontemplation, contemplation, preparation, action, and maintenance. Applying this model could be effective in the prevention and control of chronic diseases (247). Moreover, it should be noted that the motivation for treatment adherence can be influenced by factors such as the absence of adverse symptoms, family support, cost of medication, lack of resources, polypharmacy and complexity of medications, poor health literacy, and other social and financial barriers that should be investigated in different countries (248).

Moreover, over the three study periods, the pooled proportion of individuals with adequate DM control improved from 16 to 22%. These improvements in adequate DM control could be attributed to the utilization of the public health system, better access to physicians and healthcare facilities, and advancements in anti-diabetic agents.

Approximately 40% of all individuals with diabetes were undiagnosed, and only one-fifth of these individuals managed to achieve adequate blood glucose control. Early screening for diabetes is crucial, as it can lead to easier management and a reduction in subsequent chronic complications and related economic losses. Therefore, the healthcare infrastructure for diabetes screening and easy access to health services are of paramount importance (26). Additionally, there is a need to improve perceptions regarding medication adherence, as studies have shown that health insurance positively affects adherence to pharmacological treatment (243). Policy-making in this regard should be prioritized. In this regard, a systematic review examined the health system-level factors affecting diabetes awareness, treatment, adherence, and control and showed that financial constraints on patients and limited access to health services and medication were the main barriers. Task-sharing with pharmacists in care delivery and improvement of education programs led by healthcare professionals were two proposed solutions in this systematic review (249).

Overall, in the current study, awareness, treatment, and control of DM were found to be higher in women, the older adults, urban areas, and high-income countries. Consistent with previous studies, our findings indicate that women are more likely to be aware of their diabetes, receive treatment, and achieve better glycemic control than men, possibly due to their higher level of health concern compared to men (250). However, it is important to note that sociocultural factors may also influence these disparities (15). Furthermore, the older adults population tends to have higher awareness and treatment rates compared to other age groups, which may be attributed to their increased health consciousness (24). On the other hand, living in rural areas was associated with lower awareness, treatment, and adequate control of DM, likely due to limited access to healthcare services and a lack of knowledge (251). This aligns with a population-based study conducted in Chinese rural areas, which also reported increasing trends of awareness, treatment, and control with age (31). Additionally, populations in high-income countries demonstrate better awareness and management of diabetes, potentially due to higher education levels, better socioeconomic status, and improved access to treatment (252, 253). Evidence suggests that diabetes awareness increases significantly with education and academic level (15).

The low proportion of adequate DM control and the gap between diagnosis and treatment can be attributed to various factors, such as low adherence to therapeutic regimes due to multiple medications, inadequate access to healthcare services, and insufficient health insurance coverage for medications (254, 255). These factors should be considered in international and national health policy-making.

Considering the large burden of diabetes, it is of critical importance to understand diabetes management status, and this comprehensive systematic review and meta-analysis plays an important role in this regard. Moreover, assessing the changing trend of these proportions during the past decades is essential.

## Limitations and strength

This study has some limitations. First, although the term ‘treatment of diabetes’ encompasses broad concepts from lifestyle modification to medication treatment, in most of the included studies and our study, “adherence to medication” was considered diabetes treatment, which may affect treatment and control rates. Second, the included studies used different diagnostic tools, measurements, and definitions for diabetes, which may have resulted in misclassification. Additionally, the uneven distribution of studies across different countries and time periods, as well as the varying quality of some studies, may have influenced the results to some extent. Moreover, methodological variations, including sampling weight considerations, different survey analysis methods, and sampling techniques, are important limitations. To obtain more robust findings, it is suggested to use individual participant data (IPD) meta-analysis. Nevertheless, this study gathered data on diabetes awareness, treatment, and control on a worldwide scale, including a large number of studies in the analysis. Furthermore, we conducted stratified analyses to assess these proportions and their trends in various subgroups over the past 40 years.

## Conclusion

Based on the results of this systematic review and meta-analysis, despite the high level of awareness and treatment among the diabetic population, the success of treatment (control) is considerably low, particularly in low-income countries and rural areas. It is crucial to improve awareness, treatment, and control by strengthening the primary care system in all countries. The global state of diabetes management highlights the urgent need for a comprehensive intervention strategy, especially for diabetes control. Implementation of appropriate community-oriented public health policies, promotion of health education, development of simplified clinical guidelines, and regular screening and physician consultation can contribute to better diabetes management.

## Data availability statement

The original contributions presented in the study are included in the article/Supplementary material, further inquiries can be directed to the corresponding author.

## Author contributions

ESh: Data curation, Formal analysis, Investigation, Methodology, Validation, Writing – original draft, Writing – review & editing. NM: Conceptualization, Data curation, Investigation, Methodology, Supervision, Validation, Writing – original draft, Writing – review & editing. ESe: Investigation, Writing – review & editing. FB: Conceptualization, Writing – review & editing. H-SE: Conceptualization, Writing – review & editing. AS: Data curation, Formal analysis, Writing – review & editing. SD: Investigation, Writing – review & editing. DM: Writing – review & editing. MQ: Conceptualization, Formal analysis, Methodology, Supervision, Validation, Writing – review & editing.
