# Supplementary figures and images for: The worldwide trend in diabetes awareness, treatment, and control from 1985 to 2022: a systematic review and meta-analysis of 233 population-representative studies
Source: Front Public Health. 2024 May 17;12:1305304. doi: 10.3389/fpubh.2024.1305304 (PMC11140097; doi:10.3389/fpubh.2024.1305304)

1990 to 2000

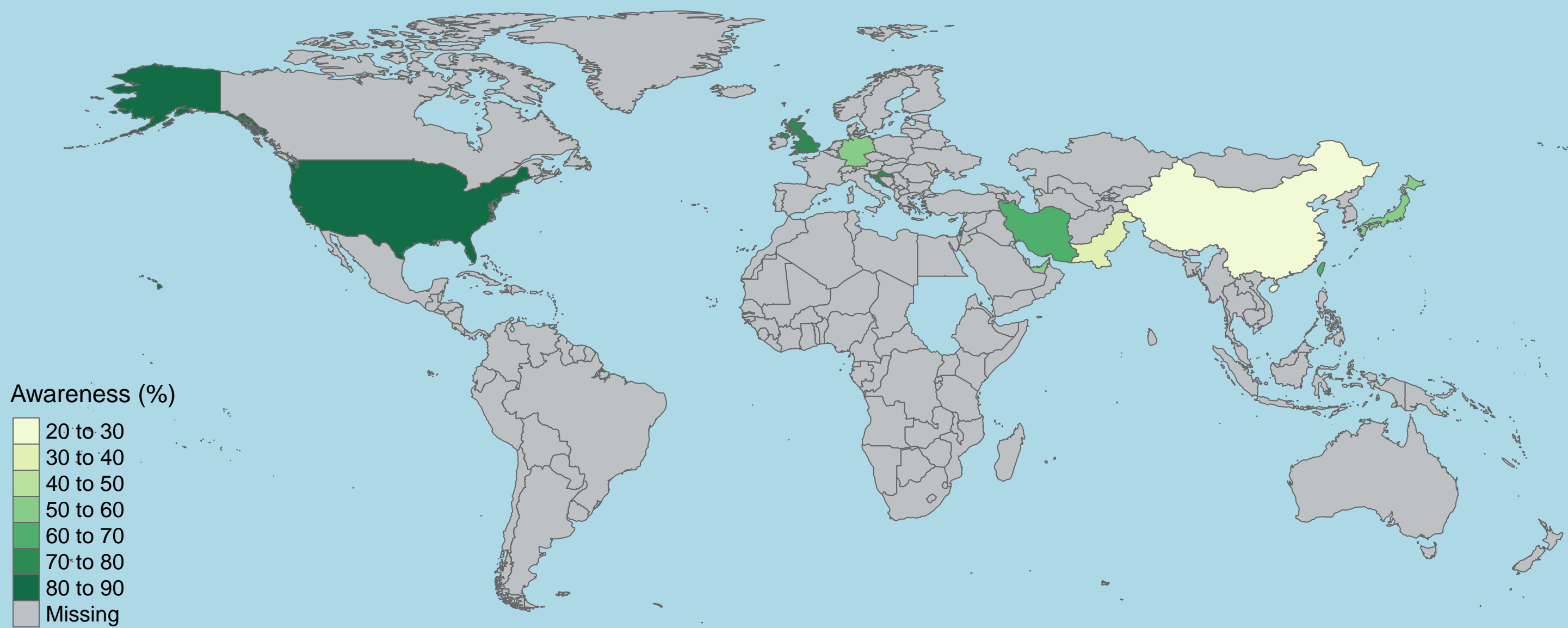

2001 to 2010

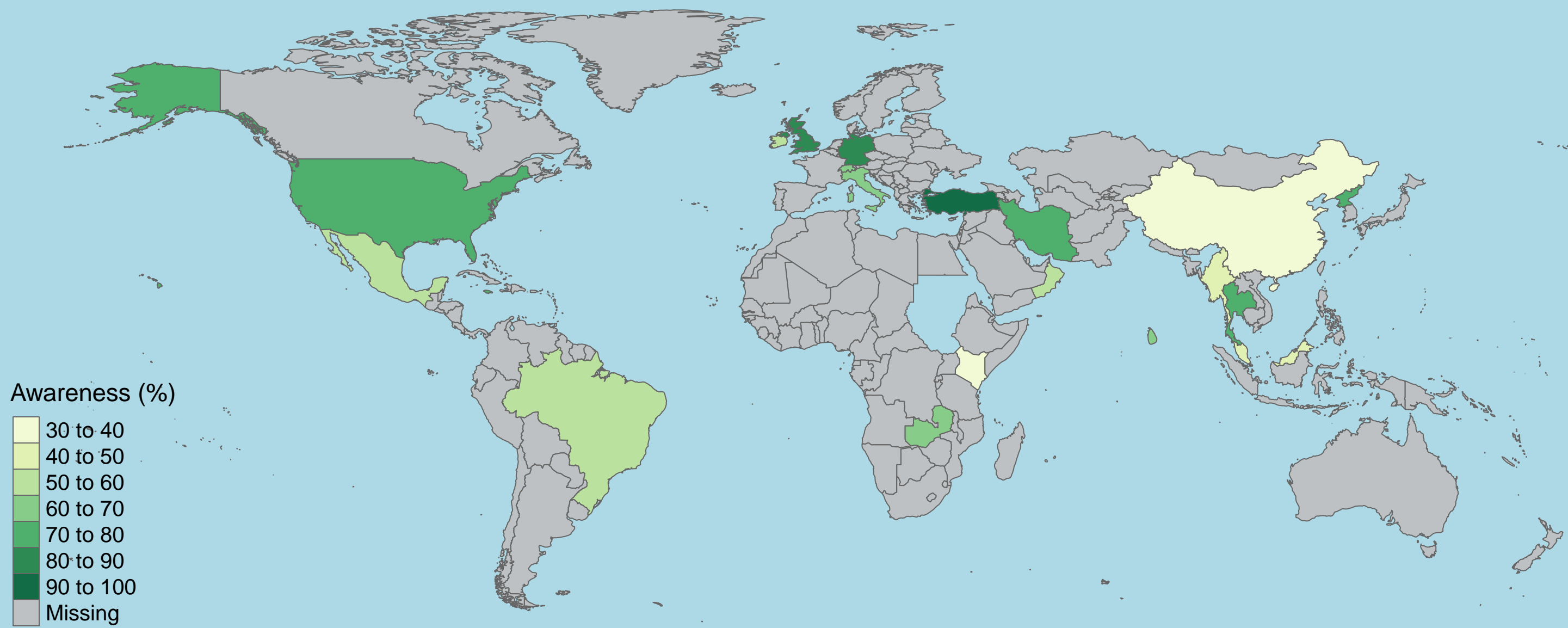

2011 to 2020

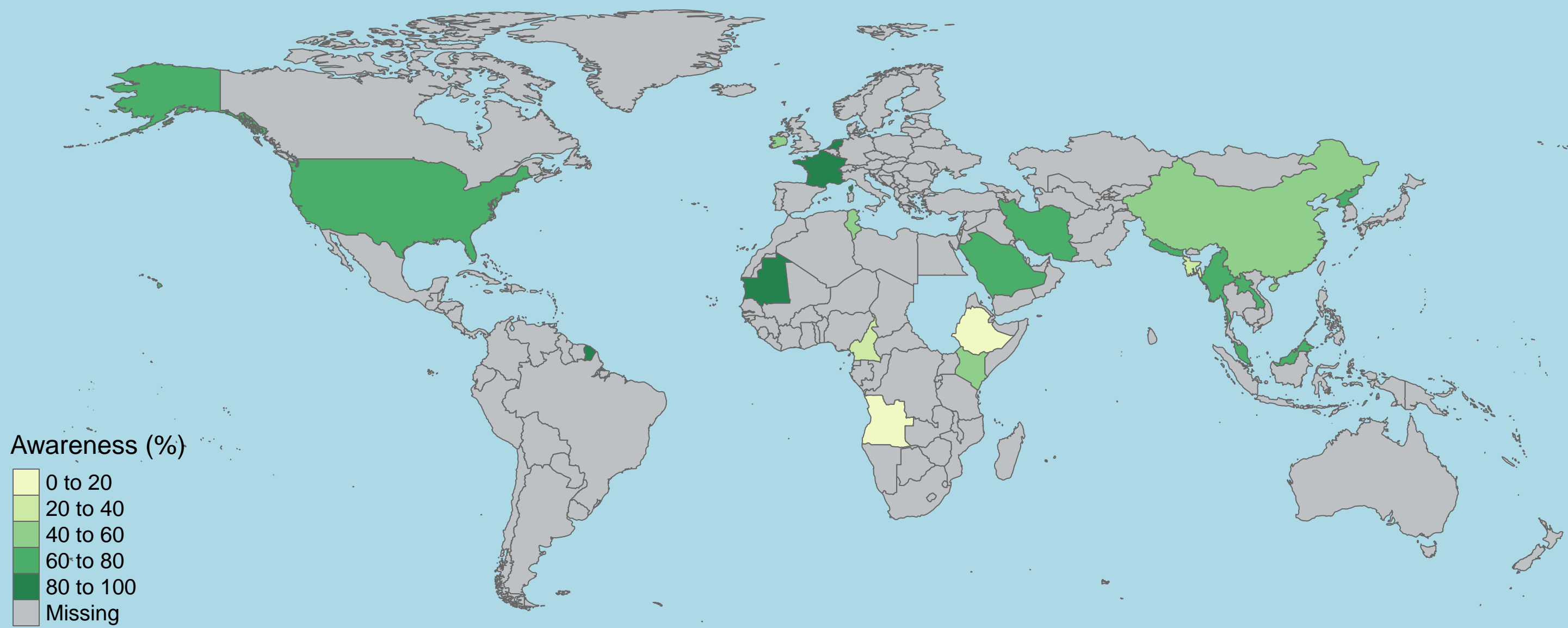

Supplement: Supplementary file 1 [file Data_Sheet_1.PDF]

1990 to 2000

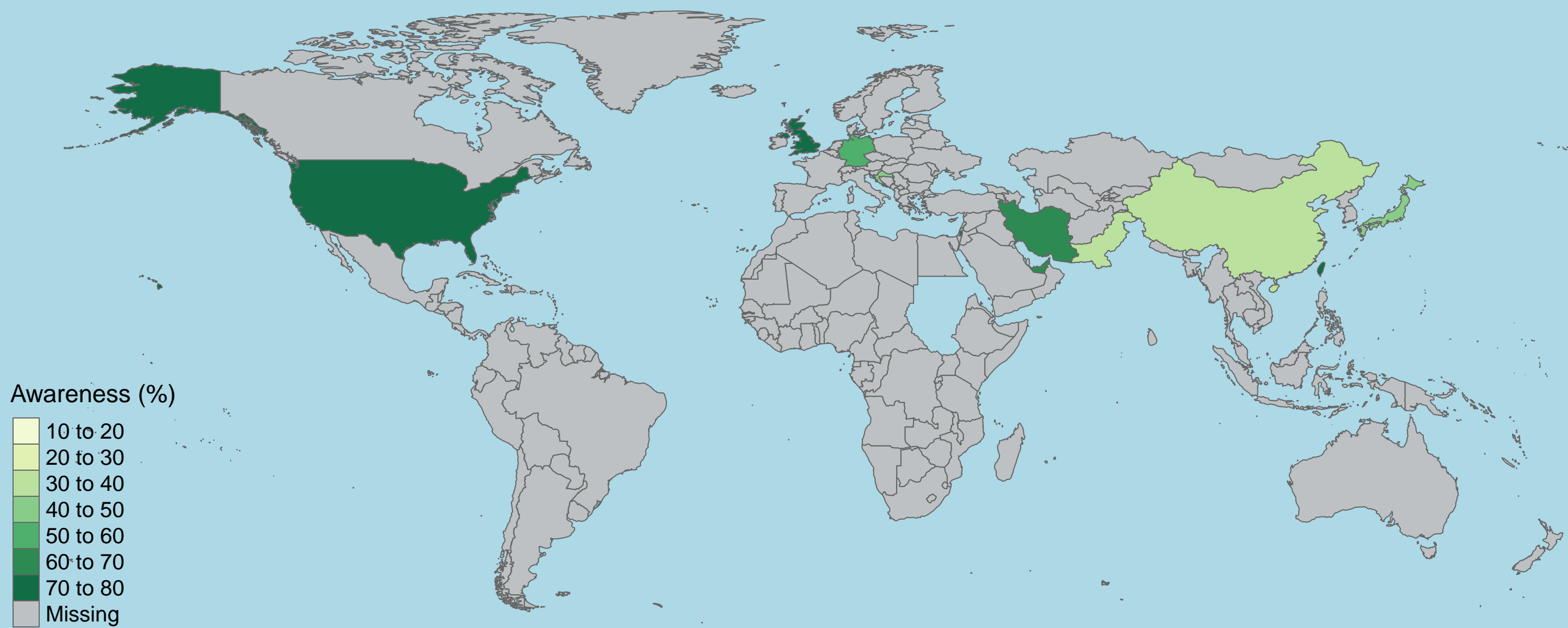

2001 to 2010

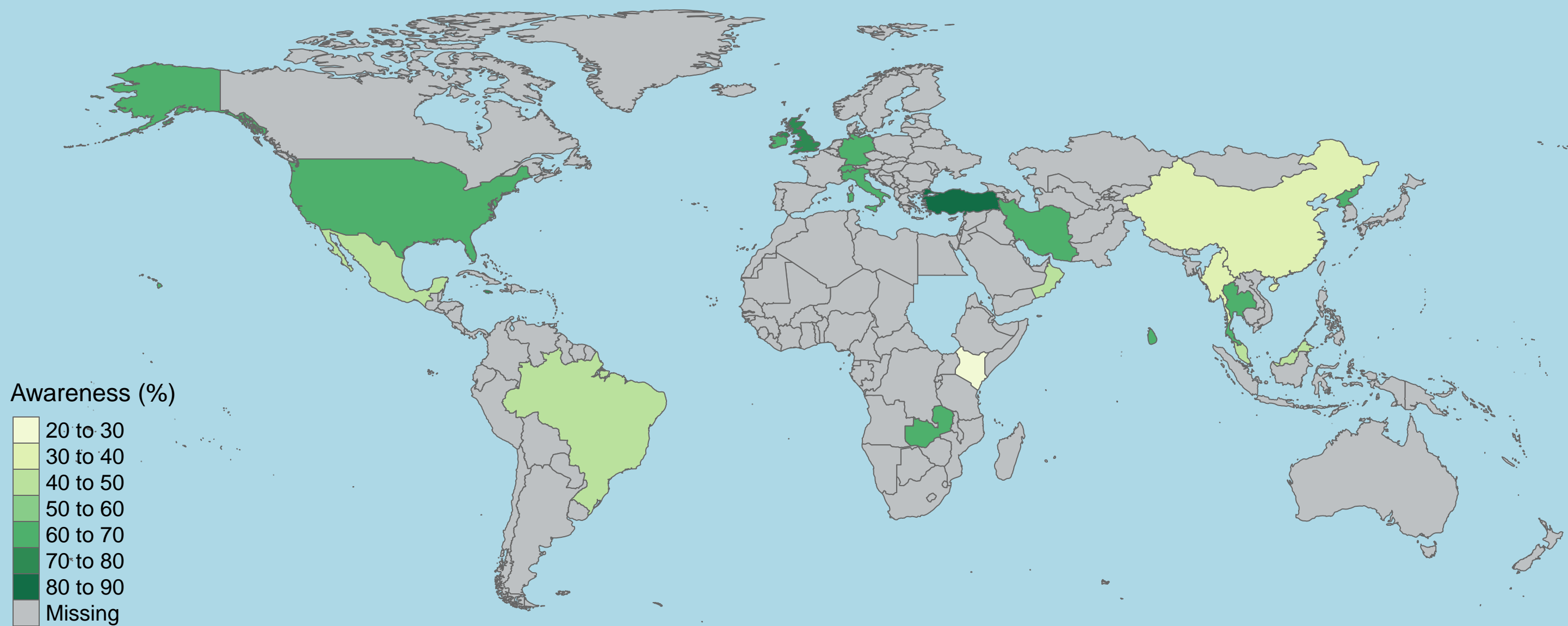

2011 to 2020

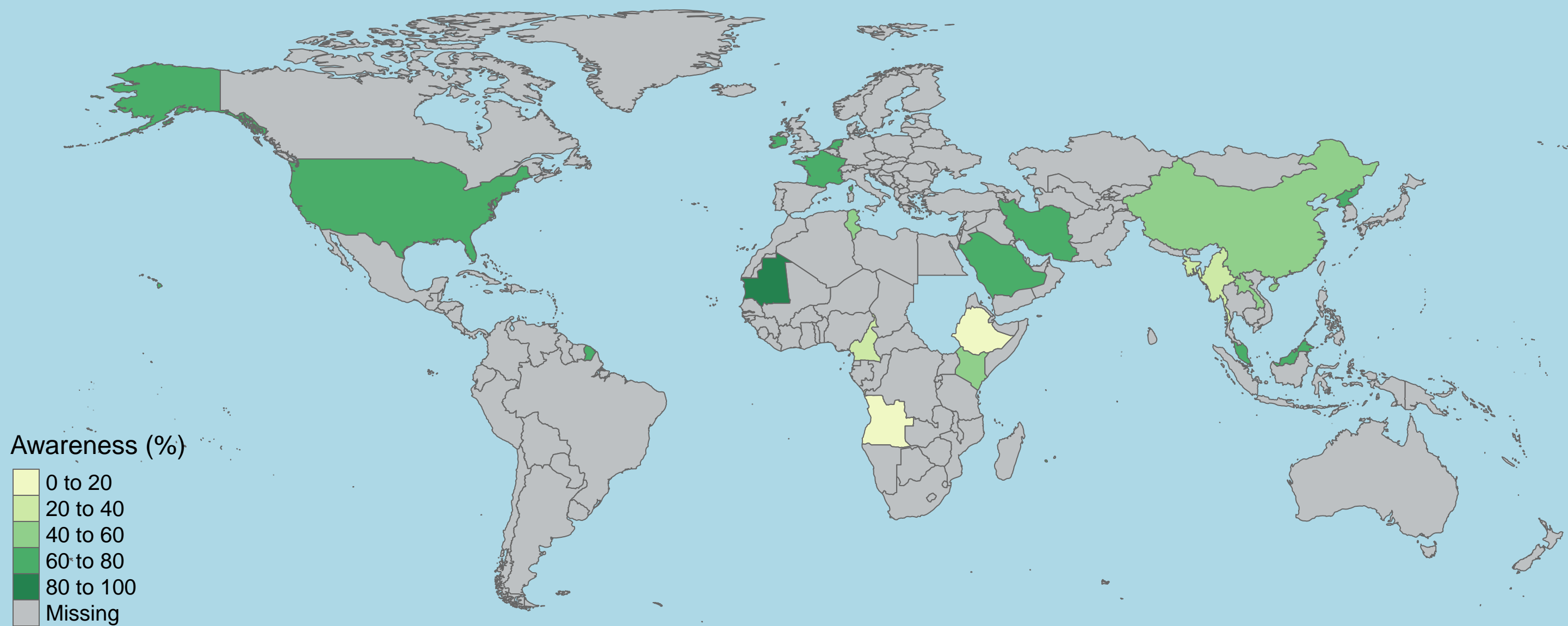

Supplement: Supplementary file 2 [file Data_Sheet_2.PDF]

1990 to 2000

Treatment (%)

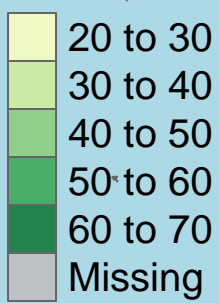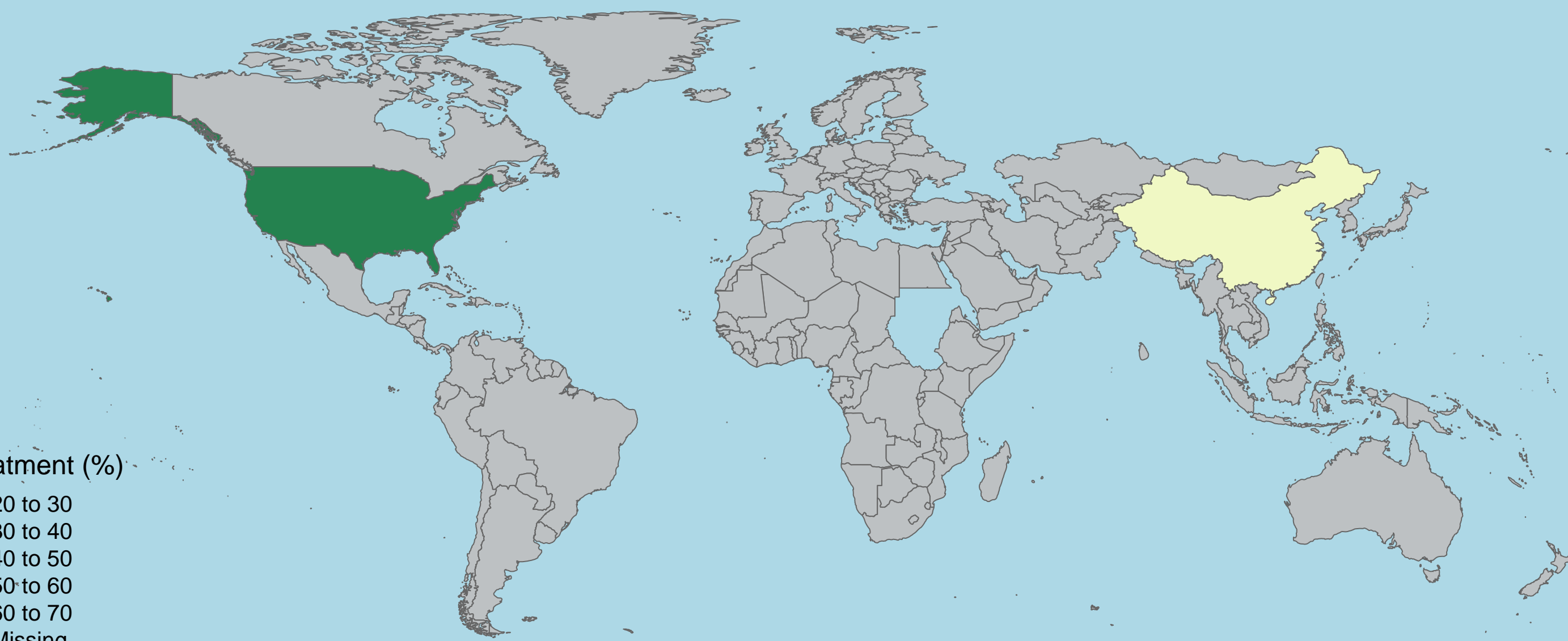

2001 to 2010

Treatment (%)

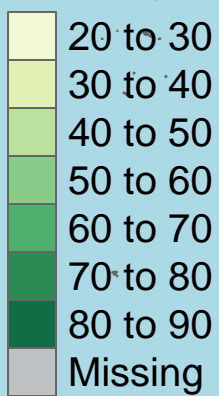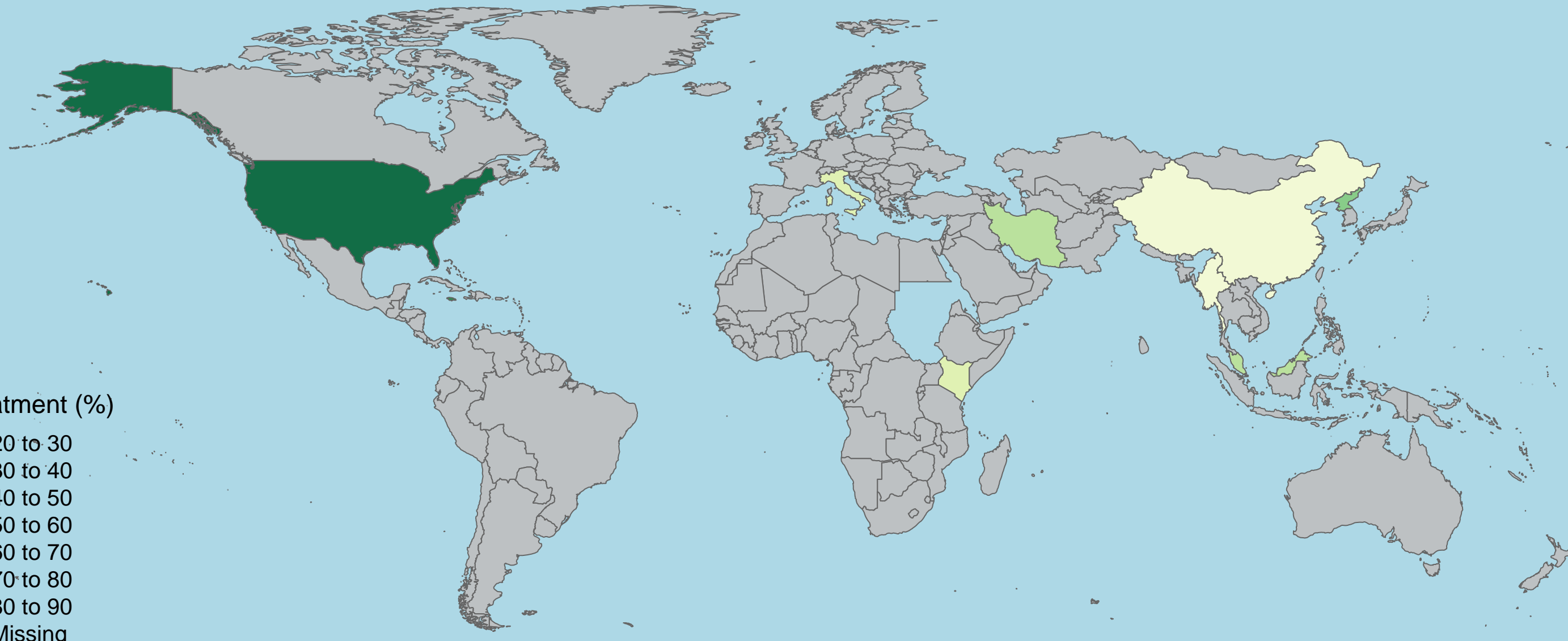

2011 to 2020

Treatment (%)

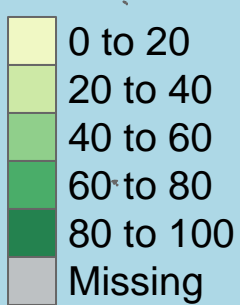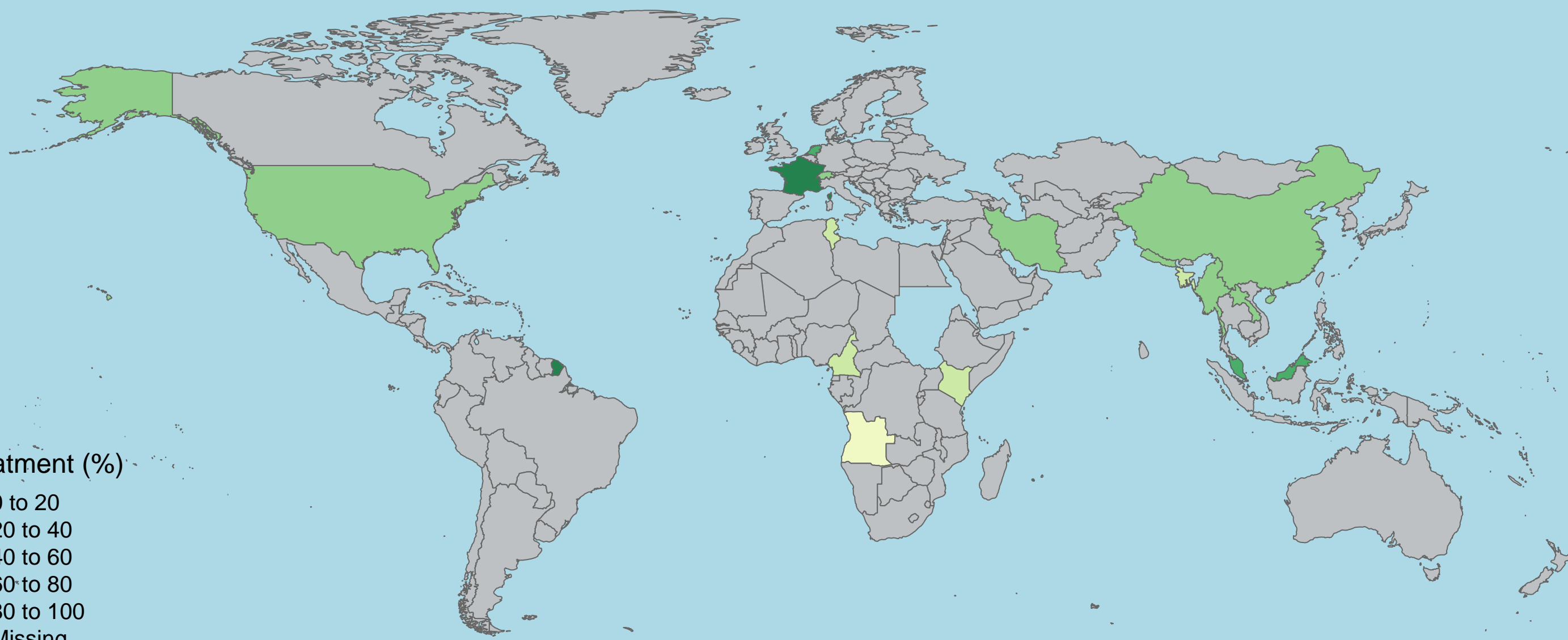

Supplement: Supplementary file 3 [file Data_Sheet_3.PDF]

1990 to 2000

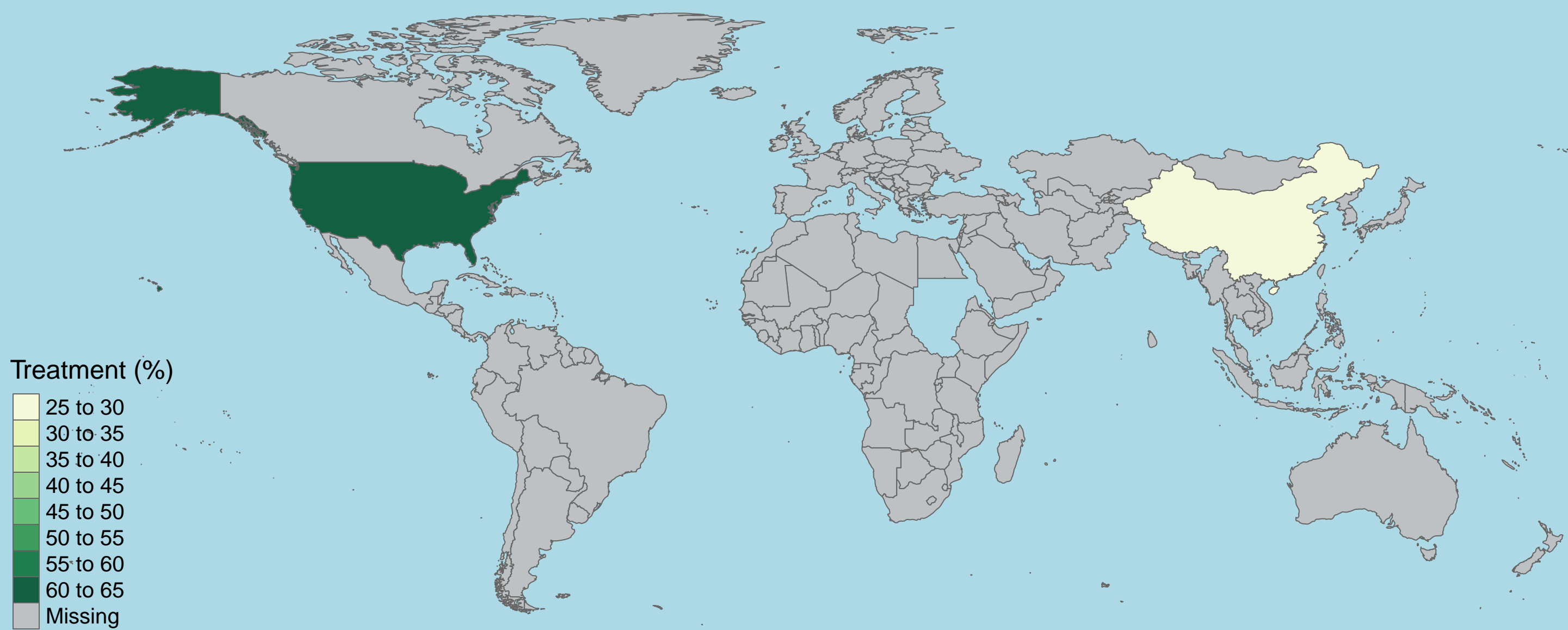

2001 to 2010

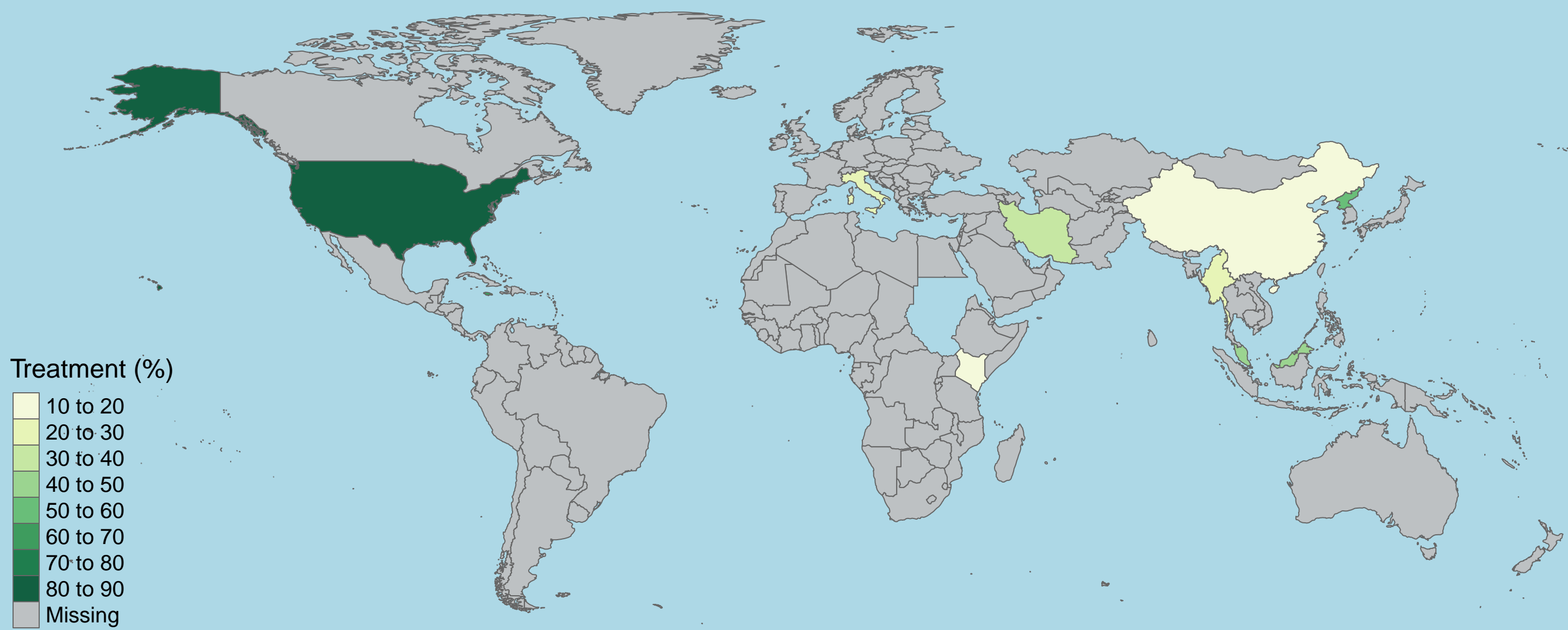

2011 to 2020

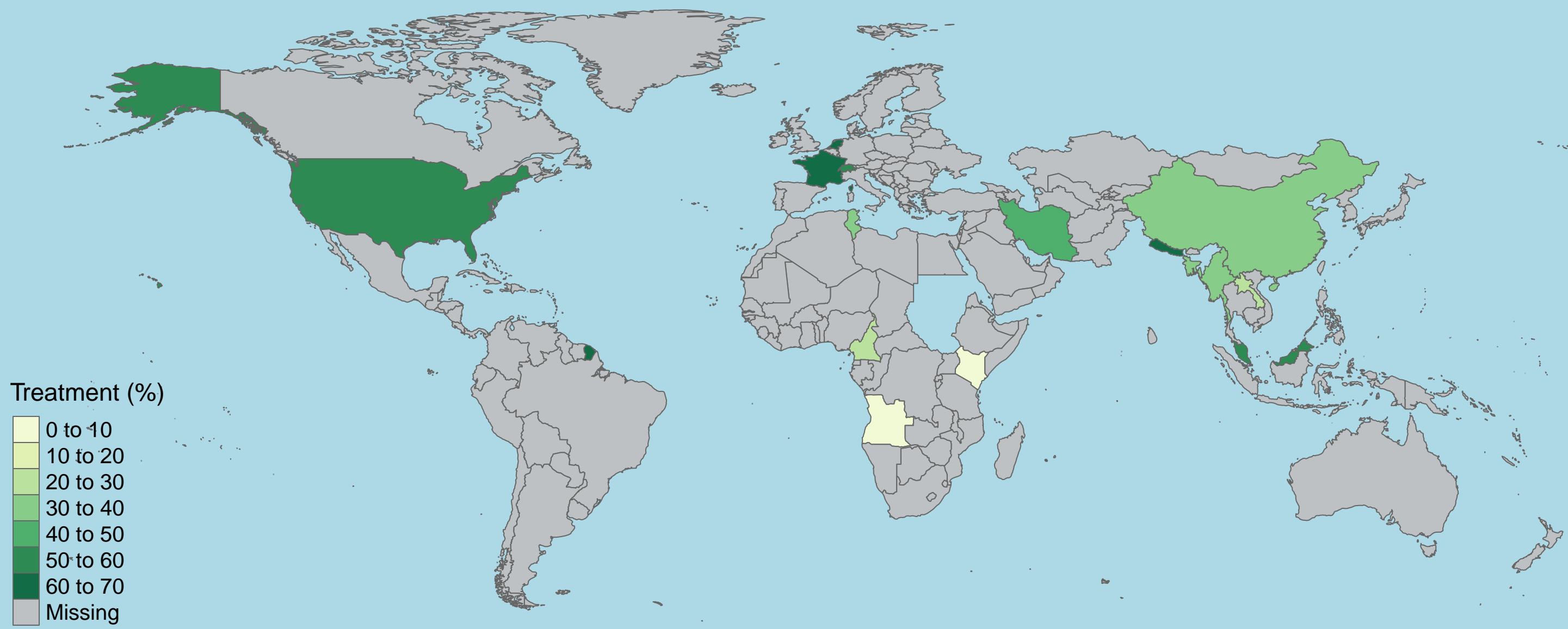

Supplement: Supplementary file 4 [file Data_Sheet_4.PDF]

1990 to 2000

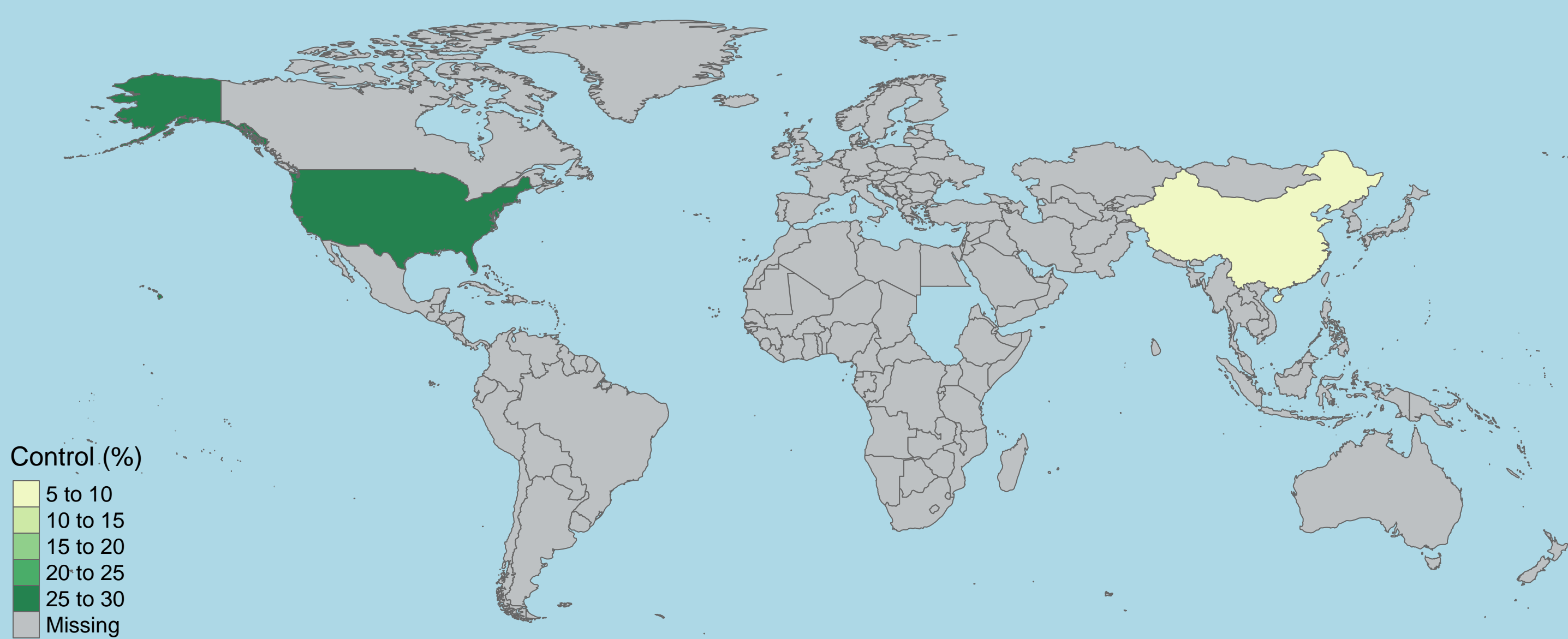

2001 to 2010

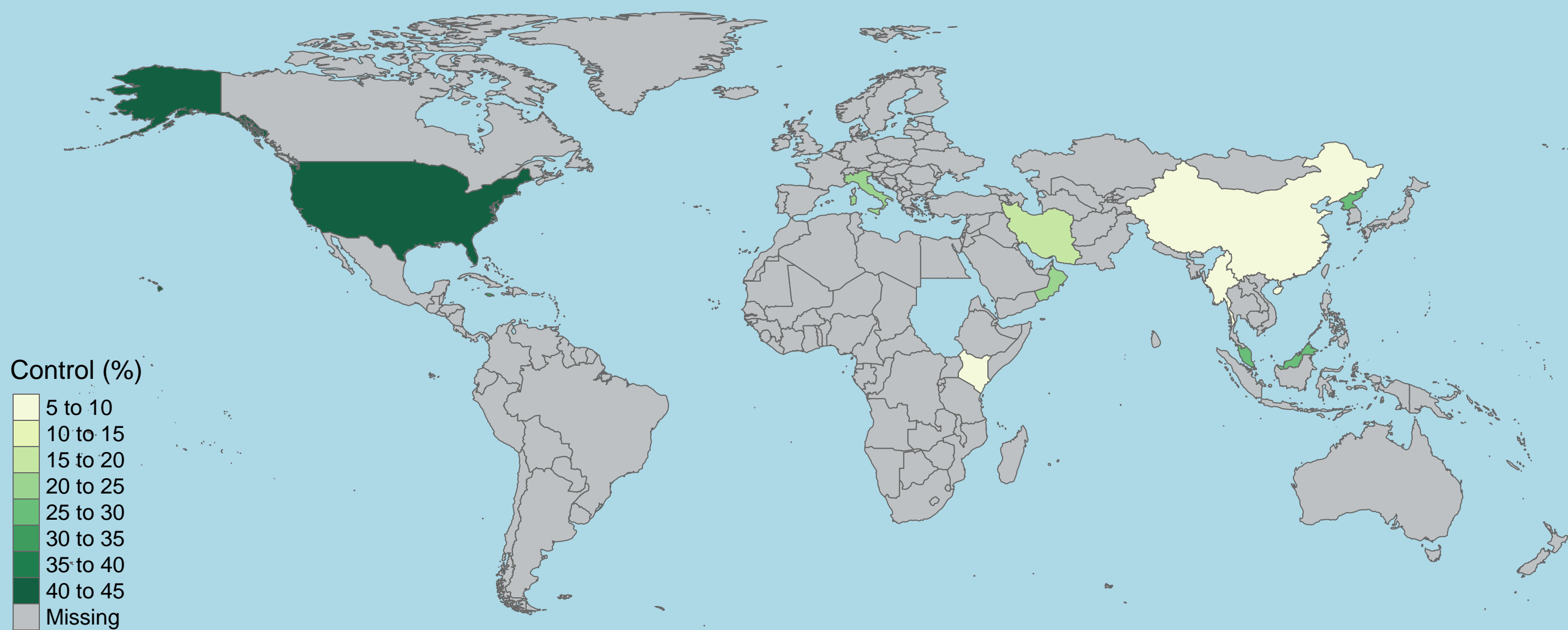

2011 to 2020

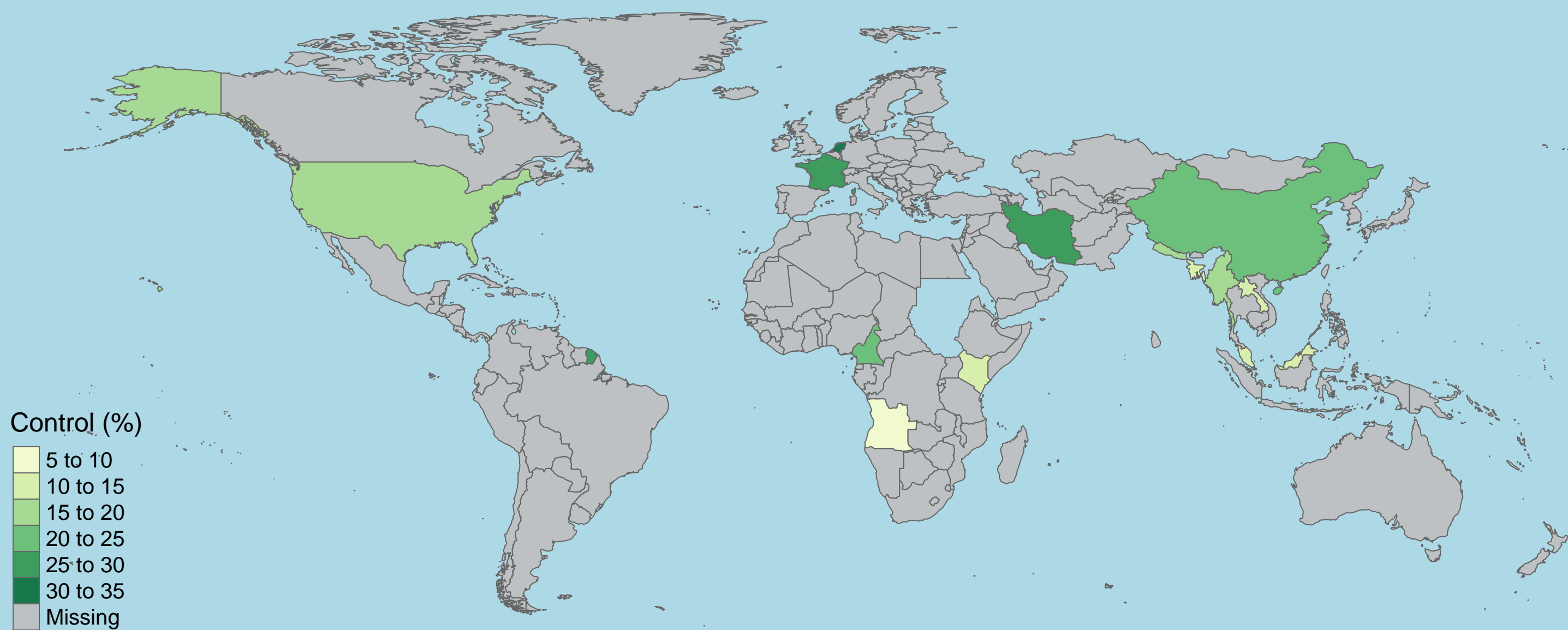

Supplement: Supplementary file 5 [file Data_Sheet_5.PDF]

1990 to 2000

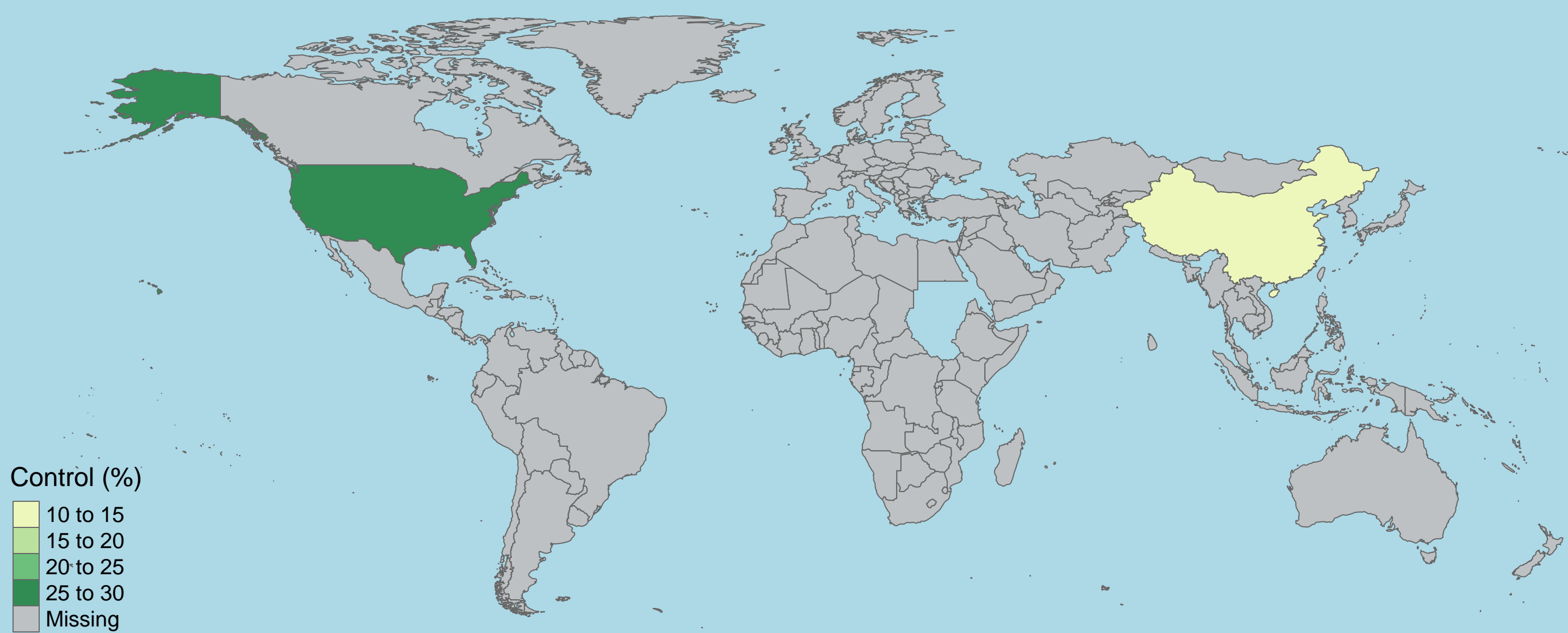

2001 to 2010

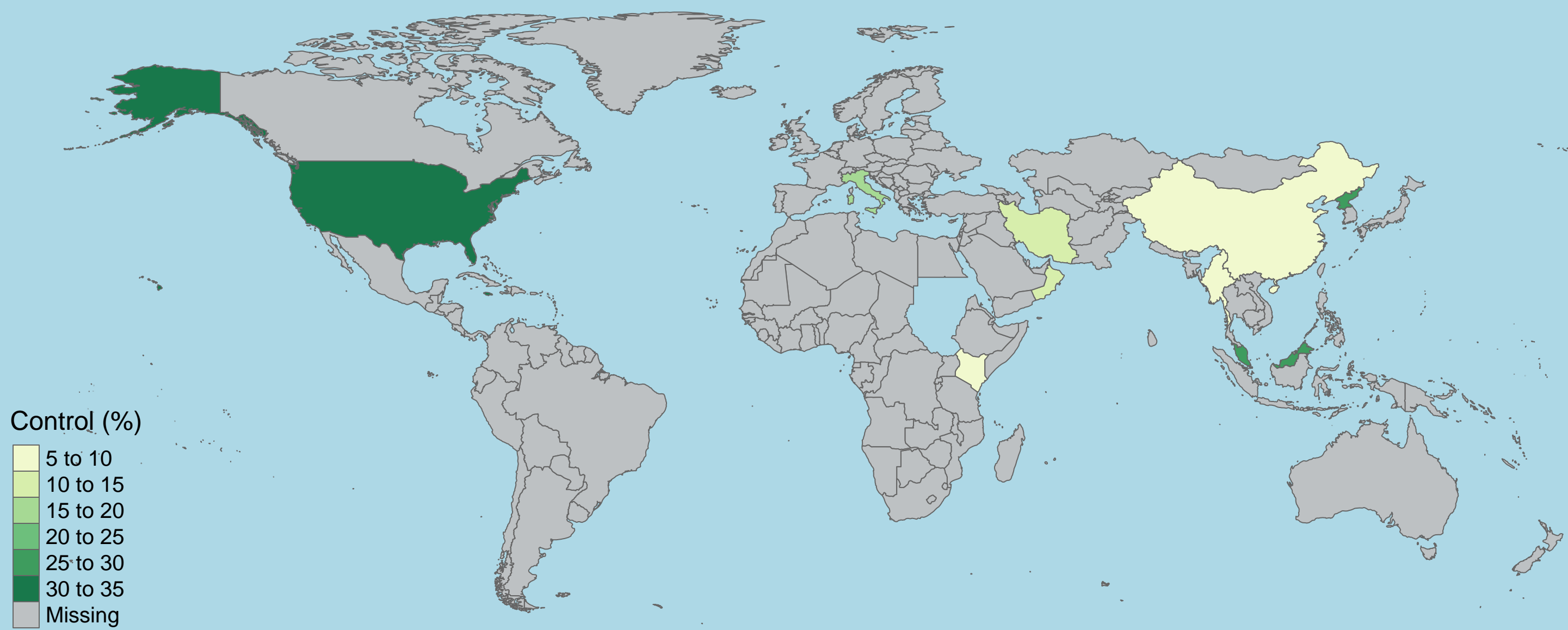

2011 to 2020

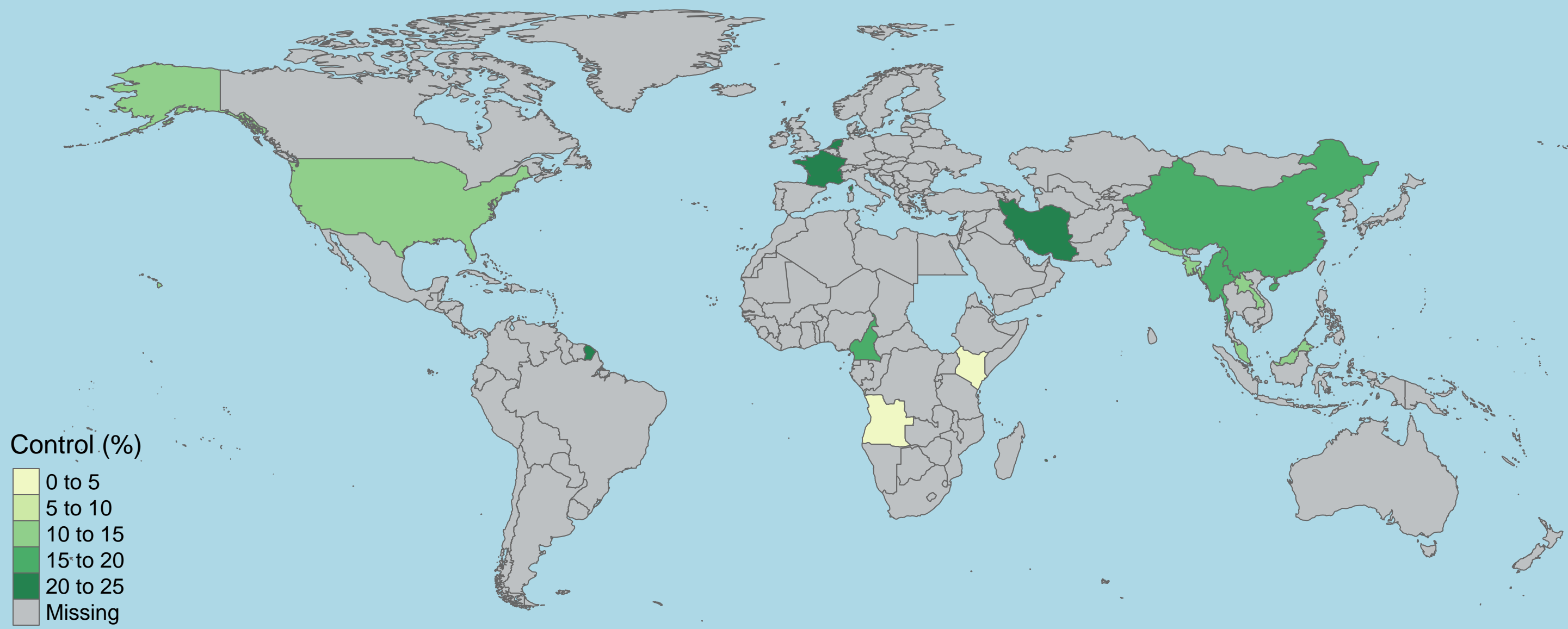

Supplement: Supplementary file 6 [file Data_Sheet_6.PDF]

1990 to 2000

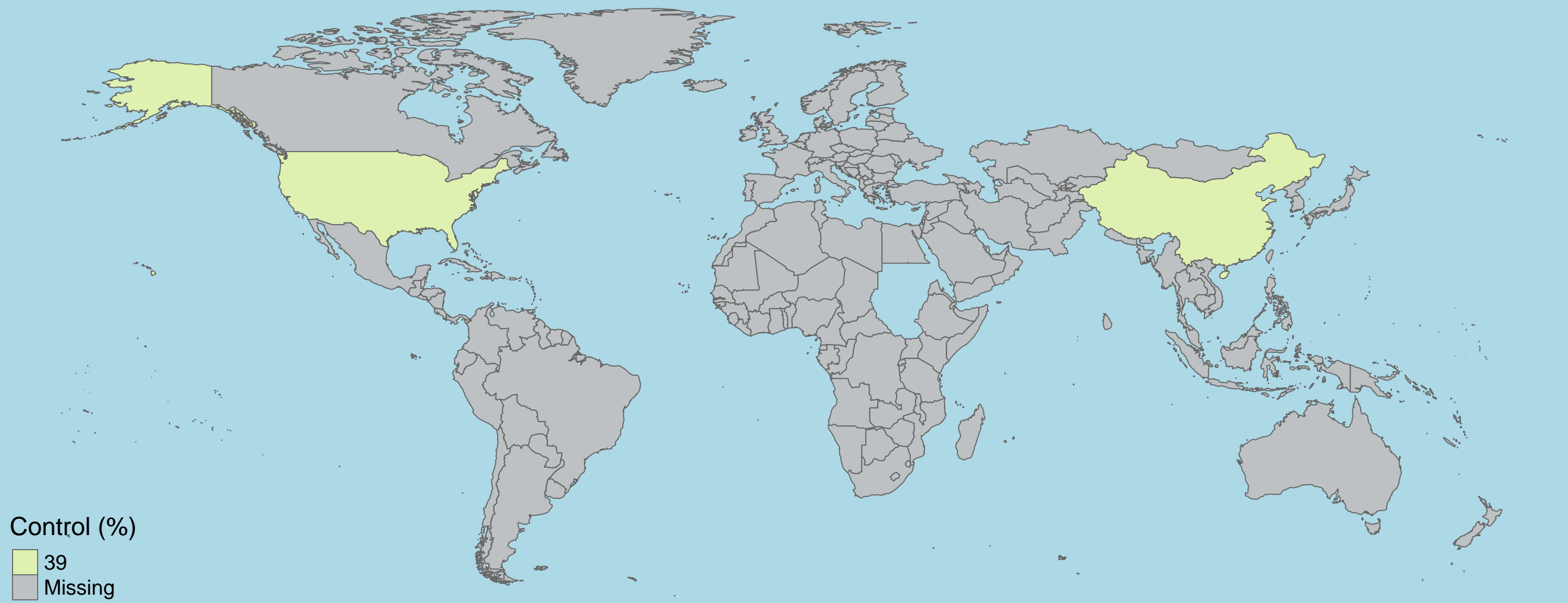

2001 to 2010

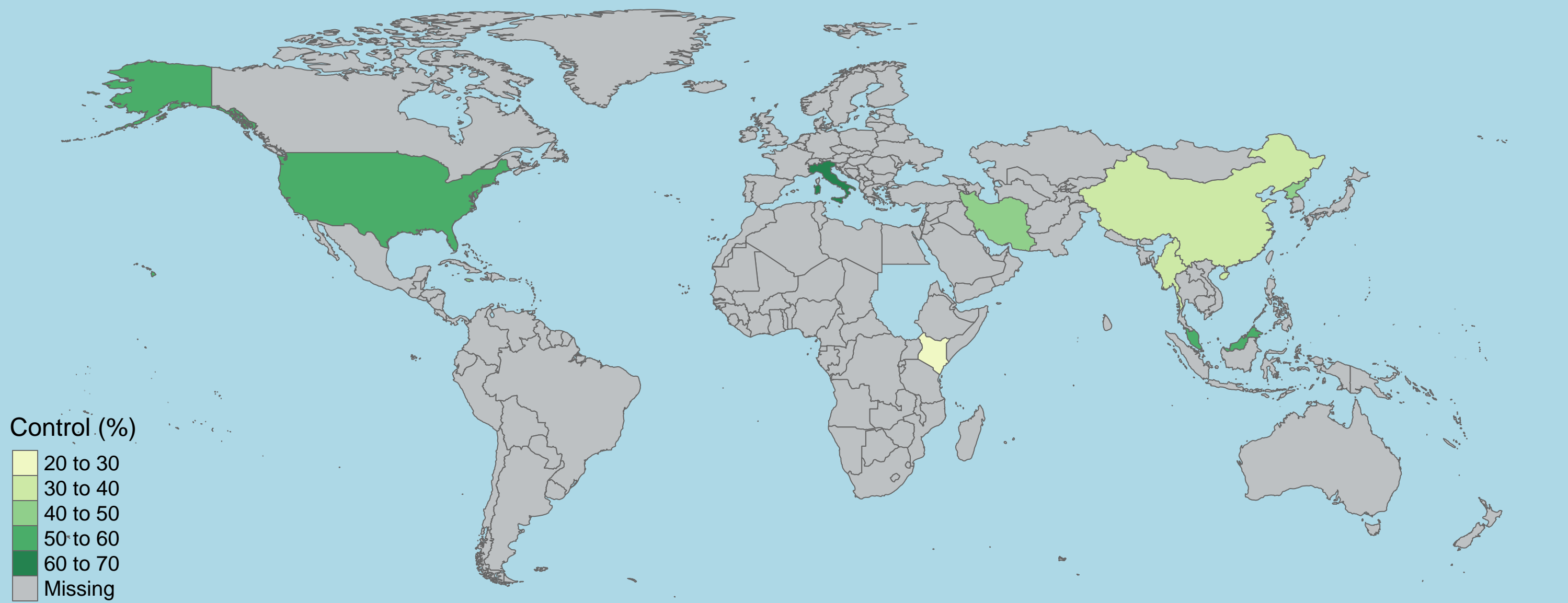

2011 to 2020

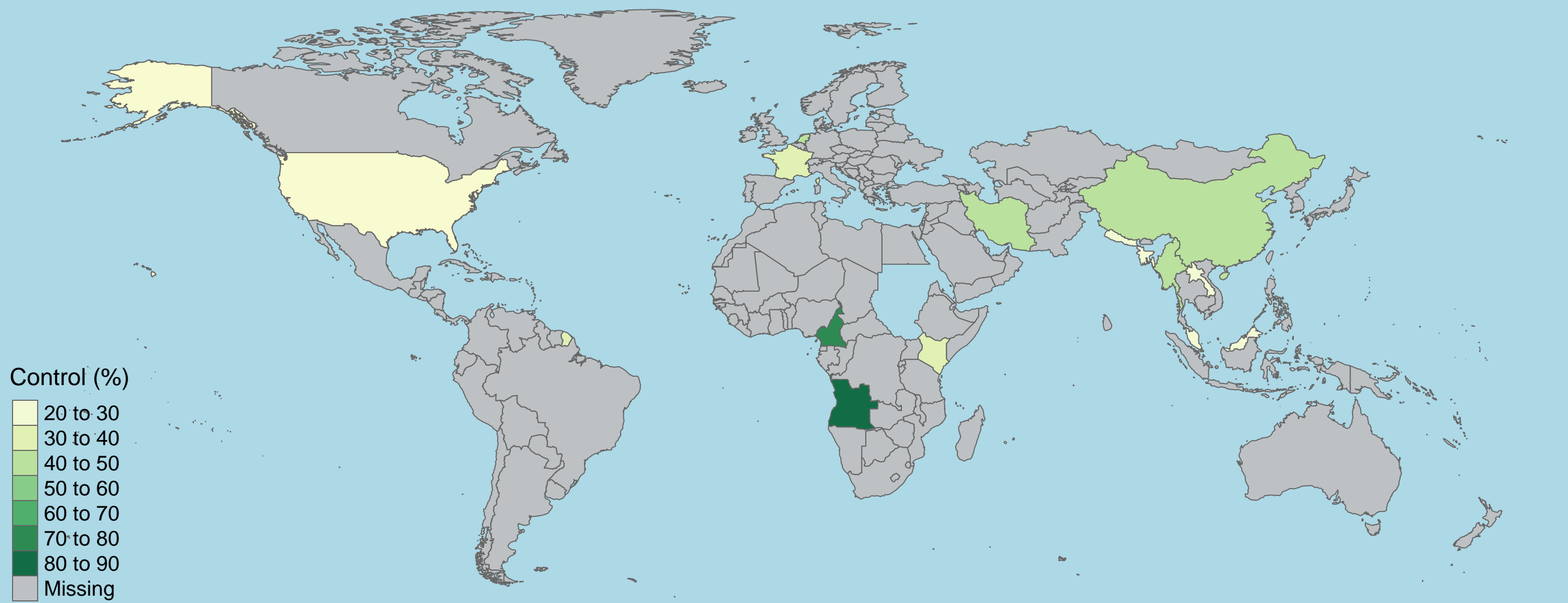

Supplement: Supplementary file 7 [file Data_Sheet_7.PDF]

1990 to 2000

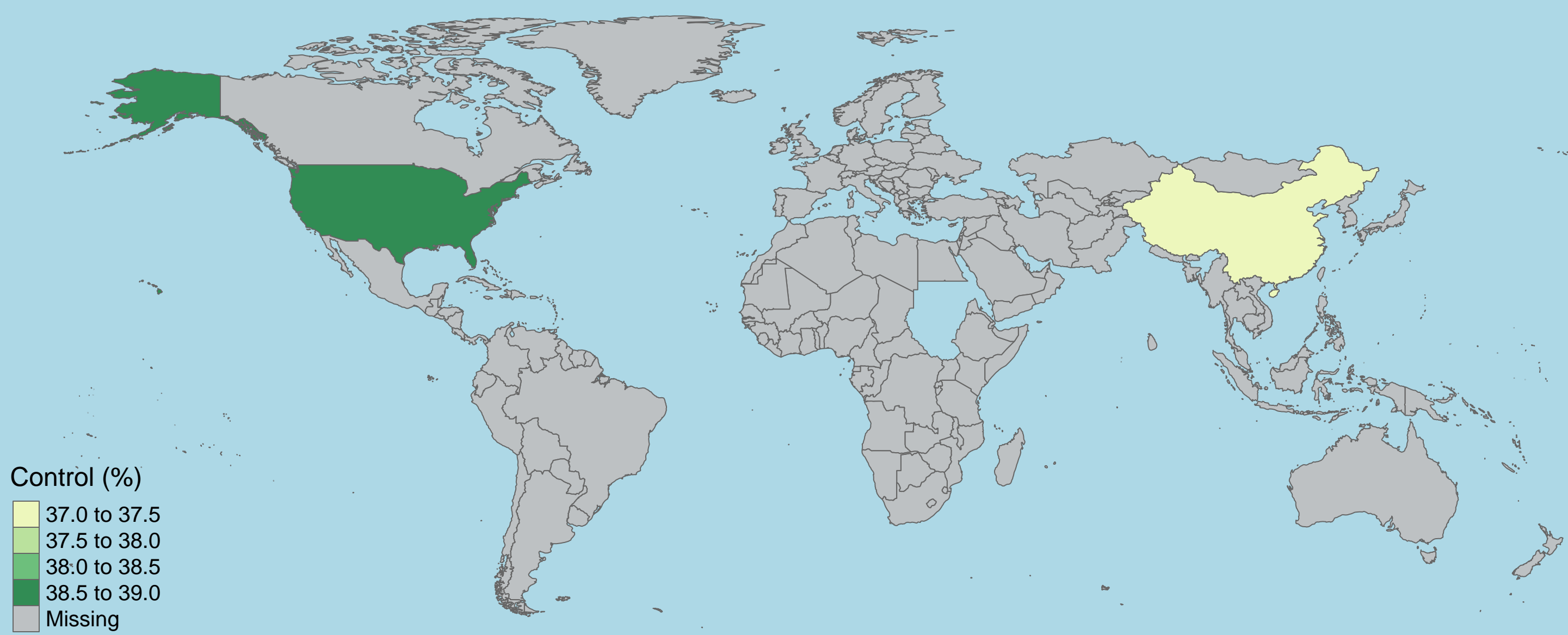

2001 to 2010

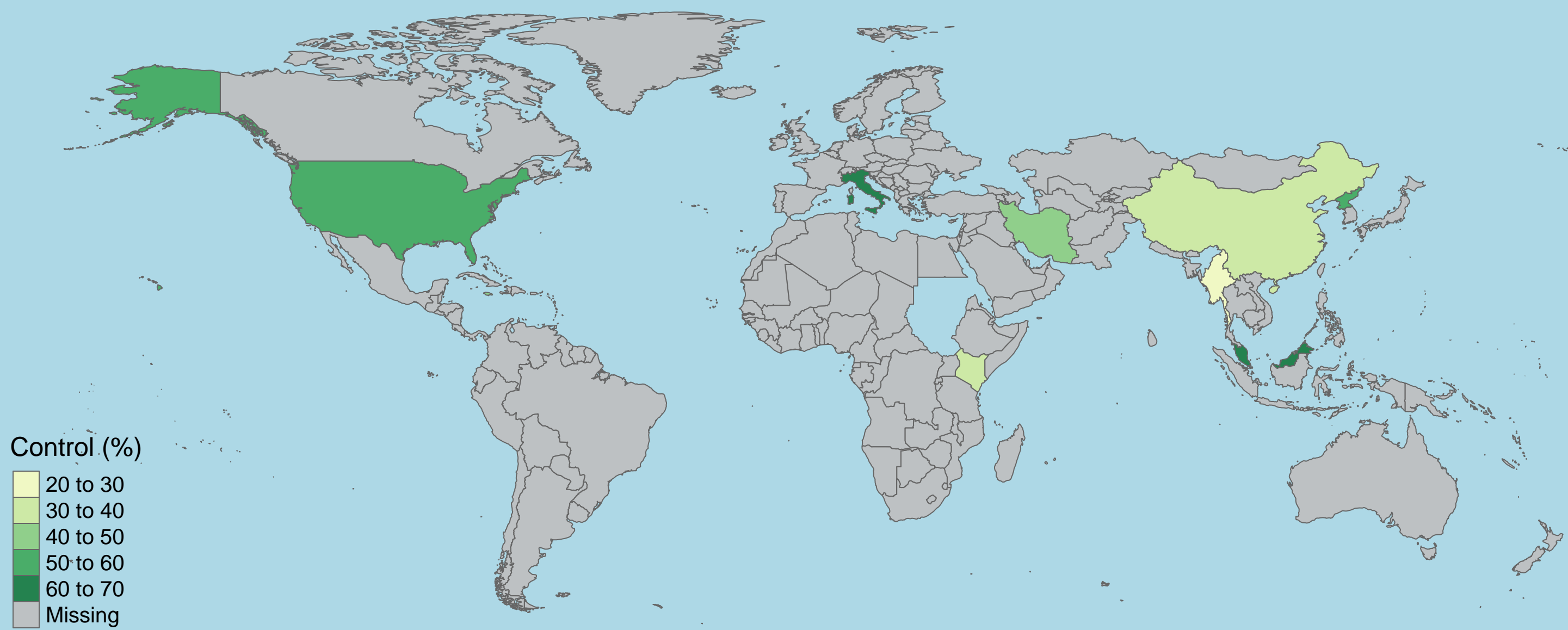

2011 to 2020

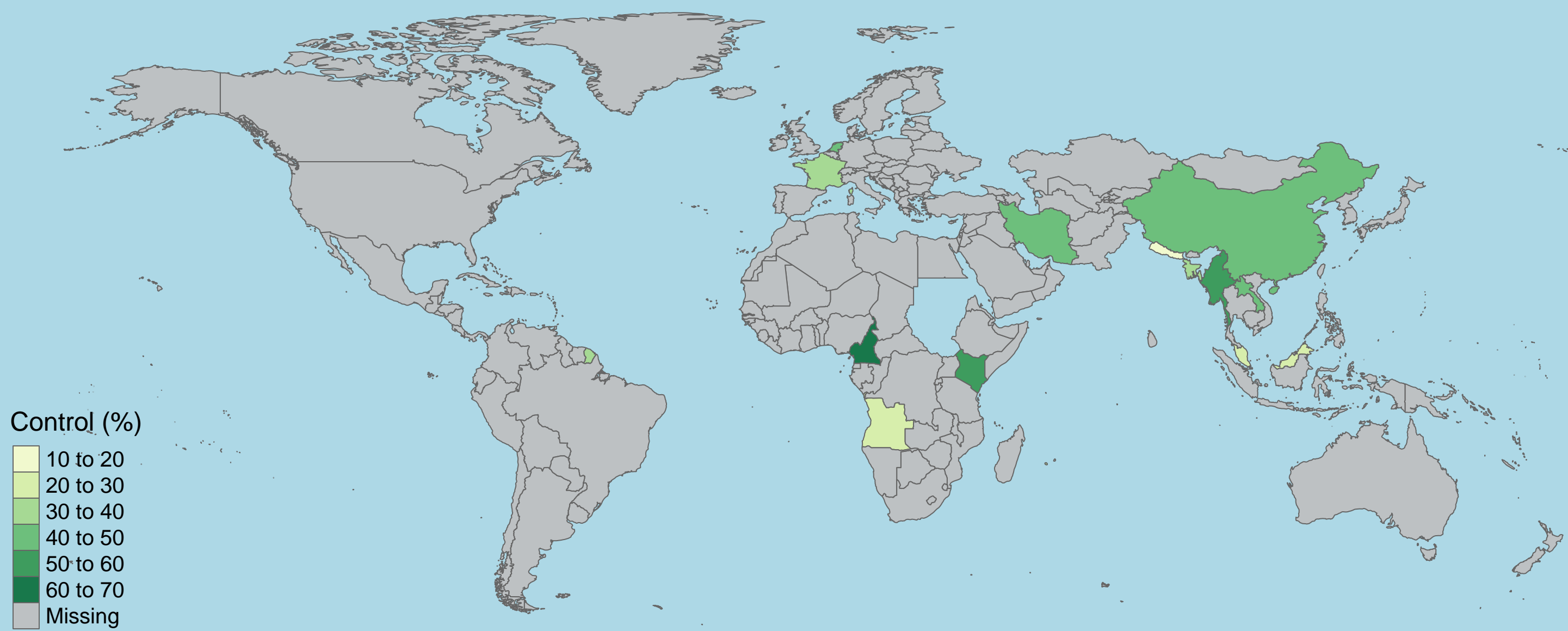

Supplement: Supplementary file 8 [file Data_Sheet_8.PDF]
