# Supplementary material for: The worldwide trend in diabetes awareness, treatment, and control from 1985 to 2022: a systematic review and meta-analysis of 233 population-representative studies
Source: Front Public Health. 2024 May 17;12:1305304. doi: 10.3389/fpubh.2024.1305304 (PMC11140097; doi:10.3389/fpubh.2024.1305304)
Supplement: Supplementary file 9 [file Table_1.DOCX]

| **Appendix table 1. Search strategy ^#^** |
| --- |
| **PubMed** |
| ((((((("Diabetes Mellitus"[Mesh]) OR " Diabetes"[Mesh]) OR " Blood Glucose "[Mesh]) OR (((((("blood sugar"[Title/Abstract]) OR "plasma glucose"[Title/Abstract]) OR hyperglycemia*[Title/Abstract]) OR "pre-diabetes"[Title/Abstract]) OR prediabetes[Title/Abstract]))) AND (("Awareness"[Mesh]) OR awareness[Title/Abstract])) AND Humans[Mesh]))  ((((("Diabetes Mellitus "[Mesh]) OR " Blood Glucose"[Mesh]) OR (((((("blood sugar "[Title/Abstract]) OR " plasma glucose "[Title/Abstract]) OR hyperglycemia* [Title/Abstract]) OR " pre-diabetes"[Title/Abstract]) OR prediabetes [Title/Abstract]))) AND (((((((Therapy[Title/Abstract]) OR treatment[Title/Abstract]) OR control[Title/Abstract]) OR ("Therapeutics"[Mesh] AND Humans[Mesh])) |
| **Scopus** |
| ( ( ( TITLE-ABS-KEY ( *diabetes mellitus*)  OR  TITLE-ABS-KEY ( *diabetes*)  OR   TITLE-ABS-KEY ( *"Blood glucose"* )  OR  TITLE-ABS-KEY ( *"blood sugar"* )  OR  TITLE-ABS-KEY ( *"FBG"* )  OR  TITLE-ABS-KEY ( *"plasma sugar"* )  OR  TITLE-ABS-KEY ( *"FPG"* )  OR   TITLE-ABS-KEY ( hyperglycemia*** )  OR  TITLE-ABS-KEY ( *"pre-diabetes"* )  OR  TITLE-ABS-KEY ( prediabetes )  OR  TITLE-ABS-KEY ( *"DM"* )    AND  ( ( TITLE-ABS-KEY ( *awareness* )  OR  TITLE-ABS-KEY ( *aware** ) ) ) )  ( ( ( TITLE-ABS-KEY ( *diabetes mellitus*)  OR  TITLE-ABS-KEY ( *diabetes*)  OR   TITLE-ABS-KEY ( *"Blood glucose"* )  OR  TITLE-ABS-KEY ( *"blood sugar"* )  OR  TITLE-ABS-KEY ( *"FBG"* )  OR  TITLE-ABS-KEY ( *"plasma sugar"* )  OR  TITLE-ABS-KEY ( *"FPG"* )  OR   TITLE-ABS-KEY ( hyperglycemia*** )  OR  TITLE-ABS-KEY ( *"pre-diabetes"* )  OR  TITLE-ABS-KEY ( prediabetes )  OR  TITLE-ABS-KEY ( *"DM"* )    AND  ( ( TITLE-ABS-KEY ( *treatment* )  OR  TITLE-ABS-KEY ( *control* )  OR  TITLE-ABS-KEY ( *treat** )  OR  TITLE-ABS-KEY ( *therapy* ) ) ) ) |
| ISI/WOS |
| TOPIC: (Diabetes Mellitus) OR TOPIC: (Diabetes) OR TOPIC: ("Blood Glucose") OR TOPIC: ("blood sugar ") OR TOPIC: ("plasma glucose")  ORTOPIC: (hyperglycemia*) OR TOPIC: ("pre-diabetes") OR TOPIC: (prediabetes) OR TOPIC: ("DM") OR TOPIC: ("FBS") OR TOPIC: ("FPG")  Indexes=SCI-EXPANDED, SSCI, CPCI-S, CPCI-SSH, ESCI Timespan=All years  TOPIC: (awareness)  Indexes=SCI-EXPANDED, SSCI, CPCI-S, CPCI-SSH, ESCI Timespan=All years  TOPIC: (Diabetes Mellitus) OR TOPIC: (Diabetes) OR TOPIC: ("Blood Glucose") OR TOPIC: ("blood sugar ") OR TOPIC: ("plasma glucose")  ORTOPIC: (hyperglycemia*) OR TOPIC: ("pre-diabetes") OR TOPIC: (prediabetes) OR TOPIC: ("DM") OR TOPIC: ("FBS") OR TOPIC: ("FPG")  Indexes=SCI-EXPANDED, SSCI, CPCI-S, CPCI-SSH, ESCI Timespan=All years  TOPIC: (tratment) OR TOPIC: (control) OR TOPIC: (therapy) OR TOPIC: (treat*)  Indexes=SCI-EXPANDED, SSCI, CPCI-S, CPCI-SSH, ESCI Timespan=All years |
