# Supplementary material for: The worldwide trend in diabetes awareness, treatment, and control from 1985 to 2022: a systematic review and meta-analysis of 233 population-representative studies
Source: Front Public Health. 2024 May 17;12:1305304. doi: 10.3389/fpubh.2024.1305304 (PMC11140097; doi:10.3389/fpubh.2024.1305304)
Supplement: Supplementary file 10 [file Table_2.DOCX]

| **Appendix table 2:** Study characteristics and reported awareness, treatment and control rate in included studies | | | | | | | | |
| --- | --- | --- | --- | --- | --- | --- | --- | --- |
| **Country** | **Study characteristic** | | | | **Prevalence** | | | **QA (10/10)** |
|  | **Author,**  **Year** | **Population**  **/study year/**  **sample size**  **/Gender/Men%/**  **setting** | **Mean age/**  **age range** | **Diabetes definition/**  **cut off** | **Awareness**  **(95% CI)** | **Treatment**  **(95% CI)** | **Control**  **(95% CI)** |  |
| China | Chaozhou Mou, 2021,  ^31^ | Population-based/  (2011-2012)/  11,587/  Both/  44.2/  Both | 59.5  >45 | FBS ≥ 126 mg/dL, or RBS ≥ 200 mg/dL, or HbA1c ≥ 6.5% | 52.4  (49.6, 55.2) | _ | _ | 6 |
|  | Anying Bai, 2021  ^32^ | Population based/  (2015)/  12,485/  Both  47.2/  Both | 60.6±9.7  >45 | (1) a self-reported previous diagnosis by healthcare professionals (2) FBS ≥ 126 mg/dL and/or HbA1c >6.5% | 39.3  (36.9, 41.7) | 18.5  (16.6, 20.5) | 10.9  (9.4, 12.5) | 8 |
|  | Cong Liu, 2020  ^33^ | Population-based/  (2019)  3,922/  Both  44.0/  Both | 58.5±10.3  >40 | FBS ≥ 126 mg/dL ; and the patient had a clear diabetic history and/or was taking hypoglycemic drugs | 82.3  (79.0, 85.2) | 36.6  (32.8, 40.6) | 17.0  (14.2, 20.3) | 7 |
|  | Zhang, 2020  ^34^ | Population based (1992)/  1,091/  Both  38.8/  Both | 35-64 | Self-reporting of a previous diabetes mellitus diagnosis made by a doctor. | 42.9  (23.0, 86.5) | 11.1  (9.2, 13.0) | 10.0  (1.2, 31.7) | 8 |
|  |  | Population based (2011)/  2,338/  Both  40.0/  Both |  |  | 44.1  (37.7, 50.2) | 20.0  (15.0, 25.4) | 9.0  (5.0, 13.2) |  |
|  | Jingyan Li, 2019  ^23^ | Population based/  (2014 and 2015)  3,725/  Both  41.2/  Rural | 60.0±9.7  >45 | FBS ≥ 126 mg/dL , a prior history of diagnosed diabetes, or requirement of insulin or oral antidiabetic drugs in the patient | 51.8  (47.4, 56.1) | 38.6  (34.5, 42.9) | 5.4  (3.7, 7.7) | 8 |
|  | Li, C, ^35^ | Population based/  (2011 and 2015)  9,357/  Both  46.1/  Both | >45 | FBS ≥ 126 mg/dL and/or HbA1c > 6.5% or higher or a self-reported doctor-diagnosed diabetes | 33.4  (31.1, 35.6) | _ | _ | 5 |
|  | Le Cai, 2017 ^36^ | Population based (2009)/  6,350/  Both  48.3/  Both | >35 | FBS ≥ 126 mg/dL or reported use of ant diabetic medications within the previous 2 weeks. | 32.5  (28.5, 37.0) | 20.2  (16.7, 24.0) | 6.1  (4.2, 8.6) | 6 |
|  |  | Population based (2016)/  6,359/  Both  49.4/  Rural |  |  | 49.3  (45.2, 53.4) | 32.1  (28.2, 35.8) | 13.5  (10.9, 16.5) |  |
|  | Zhang, F, 2018 ^37^ | Population based/  (2016)  4,052/  Both/  40.0 | 54.5±9.3 | FBS ≥ 126 mg/dL | 52.9  (47.3, 58.4) | 47.7  (42.3, 53.3) | 75.9  (68.2, 82.3) | 5 |
|  | Wang, Q, 2018 ^38^ | Population based/  (2011 and 2012)  10,851/  Both/  41.1/  Both | >40 | A self-reported pervious diagnosis by a healthcare professional (diagnosed diabetes), or FBS ≥ 126 mg/dL and/or 2-HPP ≥ 200 mg/dL | 40.3  (39.3, 41.2) | 25.3  (23.7, 27.0) | 6.7  (5.8, 7.8) | 7 |
|  | Yan, X, 2017 ^39^ | Population based/  (2015)  1,676/  Both/  47.2/  Both | 39.3±11.1/  18-70 | FBS >126 mg/dL, and/or self-reported physician-diagnosed condition, and/or participants reported drug treatment for diabetes | 51.9  (40.5, 63.1) | 39.5  (28.8, 51.0) | 29.6  (20.0, 40.8) | 5 |
|  | Wang, L, 2017  ^40^ | Population based/  (2013)  170,278/  Both/  42.7/  Both | 43.5±16.2  >18 | A self-reported pervious diagnosis by a healthcare professional (diagnosed diabetes),  or FBS ≥ 126 mg/dL, 2-HPP ≥ 200 mg/dL, or HbA1c level of ≥ 6.5% | 36.5  (34.3, 38.6) | 32.2  (30.1, 34.2) | 18.7  (18.2, 19.2) | 6 |
|  | Liu, X, 2017  ^41^ | Population based/  (2013)  14,009/  Both  35.1/  Rural | >18 | FBS ≥ 126 mg/dL or if participants self-reported that they were previously diagnosed with T2DM by a physician | 65.7  (63.5, 67.9) | 59.5  (57.2, 61.8) | 29.4  (27.3, 31.5) | 6 |
|  | Hu, M  ^42^ | Population based/  (2013)  8,150/  Both/  49.7/  Both | 41.0±16.1  >18 | Self-report previous diagnosed diabetes by a health care professional and an FBS ≥ 126 mg/dL | 52.5  (48.6, 56.4) | 48.0  (44.2, 52.0) | 19.8  (16.4, 22.3) | 8 |
|  | Feng, 2016  ^24^ | Population based/  (2011)  19,939/  Both/  49.7/  Both | 18-79 | FBS ≥ 126 mg/dL and/or history of diabetes and/or using insulin or hypoglycemic agents | 60.9  (58.7, 63.0) | 51.3  (49.1, 53.5) | 22.4  (20.6, 24.3) | 8 |
|  | Qin, Y, 2016  ^43^ | Population based/  (2007 and 2008)  3,136/  Both/  44.2/  Both | 18-80 | FBS ≥ 126 mg/dL , or HbA1c ≥ 6.5% (48 mmol/mol), or previous diagnosis by a physician | 28.0  (24.1, 32.2) | 25.8  (18.7, 33.7) | 12.4  (9.6, 15.6) | 5 |
|  | Liu, M  2016  ^20^ | Elderly based  (2010)/  2,102/  Both/  40.3/  Urban | 71.2±6.6  >60 | FBS ≥ 126 mg/dL or been diagnosed as having diabetes before, or having received drug treatment for diabetes regularly | 78.5  (74.7, 82.0) | 69.3  (65.1, 73.2) | 15.9  (12.9, 19.3) | 7 |
|  |  | Elderly based/  (2001)/  2,277  Both/  41.4/  Urban | 67.9±5.8  >60 |  | 74.3  (70.2, 78.2) | 51.1  (46.6, 55.6) | 20.1  (16.6, 24.0) |  |
|  | Yue, J, 2016, ^44^ | Population based/  (2013)  15,404/  Both/  47.3/  Urban | 54.4±15.8 | FBS ≥ 126 mg/dL | 58.3  (55.4, 61.2) | 51.9  (48.9, 54.8) | 14.1  (12.1, 16.3) | 7 |
|  | Su, R, 2016  ^45^ | Population based/  (2013 and 2014)  5,532/  Both/  48.4/  Both | >35 | FBS ≥ 126 mg/dLor self-reported previous diagnosis of diabetes by a healthcare professional in a hospital | 63.7  (57.6, 69.6) | 48.1  (41.9, 54.3) | 24.8  (19.7, 30.5) | 7 |
|  | Wang, R, 2016 ^46^ | Population based/  (2012)  9,600/  Both/  50.9/  Rural | 18-79 | FBS ≥ 126 mg/dL or self-reported use of anti-diabetic medications during the 2 weeks prior to the examination | 69.0  (65.4, 72.4) | 60.8  (57.0, 64.5) | 25.9  (22.7, 29.3) | 6 |
|  | Yang, F, 2016 ^47^ | Population based/  (2007)  16,375/  Both/  42.8/  Rural | >25 | A self-reported pervious diagnosis by a healthcare professional (diagnosed diabetes), or FBS ≥ 126 mg/dL and/or 2-HPP ≥ 200 mg/dL | 34.8  (30.9, 38.9) | 30.6  (24.2, 37.4) | 11.5  (4.7, 22.2) | 5 |
|  | Liu, X, 2016  ^26^ | Population based/  (2013 to 2015)  16,413/  Both  36.4/  Rural | 18-74 | FBS ≥ 126 mg/dLor self-reported use of insulin or anti-diabetic medications during the previous 2 weeks or participants reported a previous diagnosis of diabetes by a physician | 67.0  (64.9, 69.1) | 62.3  (60.2, 64.5) | 22.2  (20.4, 24.1) | 6 |
|  | Minghui Yin, 2016  ^48^ | Population-based/  (2011-2012)/  9,513/  Both/  45.7/  Both | 59.2  >45 | FBS ≥ 126 mg/dL and/or HbA1c ≥ 6.5% | 60.0  (58.2, 61.9) | _ | _ | 7 |
|  | Zhou, X, 2015 ^49^ | Population based/  (2012 and 2013)  11,600/  Both/  46.2/  Rural | >35 | FBS ≥ 126 mg/dL and/or being on treatment for diabetes | 43.5  (42.6, 44.4) | 36.6  (35.7, 37.5) | _ | 6 |
|  | Wang, C, 2014  ^50^ | Population based/  (2012)  18,260/  Both/  56.6/  Both | 18-79 | FBS ≥ 126 mg/dLor self-reported use of anti-diabetic medications during the 2 weeks prior to the examination | 67.6  (65.4, 69.7) | 56.7  (54.7, 59.2) | 24.0  (22.1, 26.0) | 6 |
|  | H. Zhang, 2013  ^51^ | Population-based/  (2005)/  7,315/  Both/ | >20 | FBS ≥ 126 mg/dL or 2-H PP > 200 mg/dL when no previous diabetes was present. | 58.1  (54.3, 61.9) | _ | _ | 6 |
|  | XU, S  2013  ^52^ | Population based ( Shaanxi)/  (2007 and 2008)  3,254/  Both/  41.9/  Both | >20 | FBS ≥126 mg/dl (7mmol/l), or 2-HPP >200 mg/dl, or on medications for high blood sugar | 34.9  (27.0, 42.8) | _ | _ | 6 |
|  |  | Population based (Fujian)/  (2007 and 2008)  2,672/  Both/  39.5/  Both |  |  | 42.3  (34.4, 50.2) | _ | _ |  |
|  | Yu Xu, 2013  ^53^ | Population based/  (2012)  98,658/  Both  45.7/  Both | >18 | Self-reported previous diagnosis by health care professionals, (2) FBS ≥ 126 mg/dL, (3) 2-HPP ≥ 200 mg/dL (4) HbA1c ≥ 6.5% | 30.1  (29.1, 31.1) | 25.8  (24.9, 26.8) | 10.2  (9.7, 10.8) | 9 |
|  | Zhang, Y, 2012, ^54^ | Population based/  (2002)  3,590/  Both/  45.1/  Both | 50.1±15.7 | FBS ≥ 126 mg/dL, 2-HPP of ≥ 11.1 mmol/l- HbA1c ≥ 6.5% | 12.9  (8.3, 17.6) | _ | _ | 5 |
|  | Yang, Y, 2012  ^55^ | Population based (Han)/  (2007-2010)  5,583/  Both/  48.3/  Both | 52.6±12.7 | FBS ≥ 126 mg/dL/l (≥ 126 mg/dl) or self-reported current diabetes treatment in the study | 53.0  (48.6, 57.4) | 26.7  (22.9, 30.7) | 10.4  (7.9, 13.4) | 6 |
|  |  | Population based (Uygur)/  (2007-2010)  4,620/  Both/  42.2/  Both | 50.7±13.0 |  | 35.8  (30.2, 41.6) | 7.3  (4.5, 10.9) | 3.1 (1.4, 5.8) |  |
|  |  | Population based (Kazak)/  (2007-2010)  3,919/  Both/  48.3/  Both | 48.7±11.7 |  | 23.8  (17.0, 31.6) | 6.3  (2.9, 11.6) | 1.4  (1.6, 4.9) |  |
|  | Weili Xu, 2012  ^56^ | Population-based/  (2005)  7,565/  Both  47.9%/  Urban | 46.8±14.0  >15 | FBS ≥ 126 mg/dL, or 2HPP ≥ 200 mg/dL | 55.5  (51.8, 59.1) | _ | _ | 8 |
|  | Le, C  2011  ^57^ | Population based/  (2008-2010)  10,007/  Both  46.3/  Rural | >18 | FBS ≥ 126 mg/dL, or when a participant reported using anti-diabetic medications or treating diabetes during  the previous diagnosis of diabetes by a medical doctor | 29.3  (25.9, 33.0) | 23.4  (20.2, 26.8) | _ | 7 |
|  | Wenying Yang, 2010  ^58^ | Population-based/  (2007-2008)  46,239/  Both/  39.8/  Both | >20 | FBS ≥ 126 mg/dL, 2-HPP ≥ 200 mg/dL , or both | 41.2  (39.9, 42.6) | _ | _ | 8 |
|  | Dongsheng Hu ^59^ | Population based/  15,236/  (2000-2001)  Both/  Both | 54.2±0.44 | according to the ADA ( FBS ≥ 126 mg/dL) | 28.5  (25.7, 31.4) | 24.7  (22.0, 27.5) | 9.4  (7.6, 11.4) | 6 |
|  | Huiguang Tian, 2009  ^60^ | Population-based/  (2004)/  769,792/  Both  47.3/  Rural | >35 | FBS ≥ 110 mg/dL | 7.3  (7.2, 7.6) | _ | _ | 6 |
| Sri Lanka | P. Katulanda 2008  ^61^ | Population based/  1,388/  both/  39.2/  both | ≥ 20 | FBS ≥ 126 mg/dL or OGTT ≥ 200 mg/dL | 63.9  (59.3-68.3) | _ | _ | 7 |
| India | Sunada Gupta, 2020  ^62^ | Elderly based/  374/  (2018)  Both  42.0/  Rural | 68.8±7.7  >60 | HbA1c ≥ 6.5% | 45.7  (34.6, 57.1) | 43.2  (32.2, 54.7) | 14.8  (7.9, 24.4) | 5 |
|  | Satyan m Rajbhandari,  2020  ^63^ | Population-based/  3,691/  Both/  -/  Both | >20 | Diabetes was defined either on the basis of a self-reported prior diagnosisor as undiagnosed diabetes on the basis of a RBS ≥ 200 mg/dL | 92.4  (90.5, 94.1) | _ | _ | 4 |
|  | Prenissl, J, 2019  ^64^ | Population based/  (2015 and 2016)2015  729,829/  Both/  13.4/  Both | 15-49 | RBS ≥ 200 mg/dl or reporting to have diabetes | 52.5  (50.6, 54.4) | 21.5  (21.0, 22.1) | 0.05  (0.05, 0.05) | 8 |
|  | K. Kandasamy 2018  ^65^ | Population-based/  425/  2018  /both/  50.8/rural | 35-75 | RBS or OGTT ≥ 200 mg/dl | 59.4 (52.6-65.9) |  |  | 3 |
|  | G. S. Anusuya 2018  ^66^ | Population based/  (2017)/  1,361/  both/  37.1/  both | 49.8 ± 13.3 | RBS ≥ 200 mg/dl | 68.2  (63.6-72.5) | _ | _ | 7 |
|  | Tripathy,  2017  ^67^ | Population-based/  2,465/  Both/  36/  Both | 18-69 | Individuals diagnosed by a physician and/or on antidiabetic medications and/or those who had FBS ≥ 126 mg/dL | 17.8  (12.9, 32.8) | _ | 6.3  (3.4, 10.5) | 7 |
|  | Negi, P.C, 2016  ^68^ | Population based/  3,582/  (2014)  Both/  45.6/  Rural | 43.7±12.7  20-70 | If patients were on anti-diabetic medications and or FBS ≥ 126 mg/dL | 40.8  (34.7, 47.3) | 22.7  (17.6, 28.4) | 4.4  (2.2, 7.8) | 6 |
|  | Gupta 2015  ^69^ | Population base/  6198/  Both/  55.2/  Both | 48 ± 10 | FBS ≥ 126 mg/dL | 65.5  (62.5, 68.5) | 51.3  (48.1, 54.4) | 28.2  (26.2, 30.2) | 8 |
|  | Singh, 2012, ^70^ | Elderly based/  474/  (209-2010)  Both/  51.7/  Urban | 65.0±7.2  >60 | FBS ≥126 mg/dL after an overnight fast for at least 8 hours, or if the participant was taking treatment for diabetes | 36.0  (26.0, 46.8) | 22.5  (14.3, 32.5) | 16.8  (9.7, 26.3) | 6 |
|  | Shashank R Joshi, 2012,  ^71^ | Population based/  15662  (2009-2010)/  both/  54.8/  Both | ≥18 | RBS or OGTT ≥ 200 mg/dL or FBS ≥ 126 mg/dL | 85.0  (84.4-86.3) | _ | _ | 6 |
|  | Bansal, 2009,  ^72^ | Population based/  (2000)  2,226  Both/  83.2/  Urban | 51.3±9.8 | FBS ≥126 mg/dL or 2-HPP ≥ 200 mg/dL or pharmacological treatment | 71.3  (65.5, 76.5) | _ | _ | 6 |
|  |  | Population based/  (2005)  2,684  Both/  76.8/  Urban | 40.1±12.2 |  | 67.0  (60.7, 72.2) | _ | _ |  |
|  | Abdul Hamid Zargar, 2008  ^73^ | Population-based/  3032/  Both/  _/  Both | 20-40 | FBS ≥ 126 mg/dL and/or 2-HPP ≥ 200 mg/dL | 9.5  (4.2, 17.9) | _ | _ | 4 |
|  | Menon, 2006,  ^74^ | Population-based/  (2002-2005)/  986/  Both/  _  Both | 18-80 | Subjects with fasting capillary glucose 110 mg/dl and or 2-h capillary glucose of 200 mg/dl were classified as having diabetes. Subjects were also considered to be diabetic if they reported history of diabetes diagnosed by a physician or if they were on anti-hyperglycemic agents irrespective of their blood glucose values | 67.2  (61.1, 72.9) | _ | _ | 4 |
| Iran | Khodakarami, 2022  ^75^ | Population-based/  (2004)/  70,961/  Both/  _  Both | 25-65 | FBS ≥ 126mg/dL or; 2) HbA1c ≥ 6.4% mmol/L or; 3) or self-report of the previous diagnosis of diabetes by medical professionals or taking medicine at the time of survey (oral glycemic medications in the last two weeks, insulin in the last two weeks) at the time of the survey. Te questionnaire did not specify the type of diabetes (1 or 2) | 53.5  (51.8, 55.2) | 35.9  (34.3, 37.5) | 14.5  (13.4, 15.6) | 8 |
|  |  | Population-based/  (2007)/  23,942/  Both/  _  Both |  |  | 65.6  (62.3, 68.8) | 42.0  (37.8, 45.3) | 20.8  (18.2, 23.5) |  |
|  |  | Population-based/  (2011)/  7,953/  Both/  _  Both |  |  | 70.5  (65.4, 75.6) | 46.0  (41.1, 50.9) | 20.4  (16.5, 24.3) |  |
|  |  | Population-based/  (2016)/  23,734/  Both/  _  Both |  |  | 82.2  (80.3, 84.1) | 39.6  (37.1, 42.1) | 18.5  (16.5, 20.4) |  |
|  | Mohammad E. Khamseh, 2021  ^76^ | Population based/  163,770/  (2014-2020)  Both/  Both | 35.70 | FBS ≥ 126 mg/dL, according to the American Diabetes Association (ADA) 2020 criteria | 79.6  (76.2, 82.9) | _ | 33.7  (33.1, 34.3) | 8 |
|  | Masoud Mirzaei,  2020,  ^77^ | Population-based/  3,810/  (2014-2015)/  Both/  46.4/  Both | 20-69 | FBS ≥ 126 mg/dL | 80.0  (77.0, 83.0) | _ | 33.5  (30.1, 36.9) | 7 |
|  | Shamshirgaran 2020  ^78^ | Population base/  2014/  1038/  Both/  45/  Urban | 52.41±11.67 | FBS > 125 mg/dL or HbA1c > 6.4% | 54.5 (47.1-61.7) | 39.1 (32.1-46.5) | 20.1 (14.6-26.5) | 9 |
|  | Roya Safari-Faramani, 2019  ^79^ | Population based/  9,999/  (2014-2017)  Both/  48.0/  Both | 47.3±9.0  35-65 | FBS of ≥126 mg/dL and/or being on diabetes medication and/or if the diabetes was confirmed by a health practitioner | 75.0  (71.9, 77.8) | 74.7  (71.2, 78.0) | 32.7  (28.5, 37.1) | 7 |
|  | N. Mahtab 2017  ^80^ | Population-based/  3,976/  both/  urban | 20-70 | The 1991 criteria of the American Diabetes Association | 34.6  (29.7-39.8) | _ | _ | 6 |
|  |  | Population-based/  4,941/  both/  urban |  |  | 56.3  (52.1-60.4) | _ | _ |  |
|  | Yazdanpanah, L, 2016 ^81^ | Population based/  ( NR)  944/  Both/  49.0/  Urban | >20 | FBS ≥ 126 mg/dL and/or oral hypoglycemic treatment and/or insulin consumption | 40.4  (31.77, 48.38) | _ | _ | 5 |
|  | Mohtasham 2015  ^82^ | Population base/  2076/  Both/  46.4/  Urban | 39.6 ± 16 | FBS ≥ 126 mg/dL | 78.29  (72.7, 83.1) | 58.14  (51.8, 64.2) | 22.87  (17.8, 28.4) | 6 |
|  | S. Khalilzadeh 2015  ^83^ | Population-based/  403/  both/  38/  both | 30-88 56.9 ± 12.8 | FBS ≥126 mg/dL  OGTT ≥ 200  previous diagnosis or oral antidiabetic treatment | 26.0  (21.8-30.6) | _ | _ | 8 |
|  | M. Katibeh 2015  ^84^ | Population-based/  2006/  2,090/  both/  47.5/  both | 40-80 | FBS ≥ 126 mg/dL | 89.6  (86.7-92.1) | _ | _ | 7 |
|  | M. H. Lotfi 2013 ^85^ | Population-based/  11027/  2012/  both/  55.7/  urban | ≥ 30 | FBS ≥ 126 mg/dL | 90.6  (89.2-91.9) | _ | _ | 8 |
|  | G. Veghari 2010  ^86^ | Population-based/  1999/  both/  50/  both | 25-65 | FBS ≥ 126 mg/dL | 74.7  (67.4-81.1) | _ | _ | 7 |
|  | Shirani, S, 2009  ^87^ | Population based/  (2000-2001)  12,514/  Both/  48.92/  Urban | 38.9±14.9 | FBS ≥ 126 mg/dL, or a 2-HPP ≥ 200 mg/dL | 54.6  (50.8, 58.3) | 46.2  (42.2, 49.7) | _ | 5 |
|  | F. Hadaegh 2008  ^88^ | Population-based /  (1999-2001)/  9,489/  both/  42.2/  urban | 43.5 ± 14.5 | FBS ≥126 mg/dL or 2-HPP ≥ 200 mg/dL | 63.3  (62.7-67.9) | 32.6 (30.1-35.2) | _ | 8 |
|  | F. Sajjadi 2008  ^89^ | Population-based/  3,940/  both/  48.6/  urban | ≥ 19 38.7 ± 0.31 | FBS ≥ 126 mg/dL  previous diagnosis or oral antidiabetic treatment | 81.4  (75.5-86.4) | _ | _ | 7 |
|  | M. Sadeghi 2007  ^90^ | Population-based/  (1999-2006)/  12,524/  both/  48.9/  urban | ≥ 19 | 2-HPP ≥ 200 mg/dL  FBS ≥ 126 mg/dL | 63.5  (60.0-66.9) | _ | _ | 9 |
| Bangladesh | Khan, 2021  ^91^ | Population base/  2017/  1174/  Both/  45.8/  Both | 46.7± 0.46 | FBS ≥ 126 mg/dL | 30.9  (28.2, 33.6) | 28.2  (25.6, 30.7) | 26.5  (19.5, 33.5) | 10 |
|  | Chowdhury, 2015 ^92^ | Population based/  (2011)  7,453/  Both  49.3/  Both | >35 | FBS ≥126 mg/dL or taking medication to reduce the blood sugar | 25.0  (24.0, 26.0) | _ | _ | 6 |
|  | Rahman, M  2015 ^93^ | Population based/  (2011)  7,540/  Both/  49.3/  Both | >35 | FBS ≥126 mg/dL or use of anti-diabetic medication | 39.7  (36.3, 43.4) | 35.5  (32.2, 38.9) | 12.9  (10.6, 15.4) | 4 |
|  | Rahman, M.S  2015  ^94^ | Population based/  (2011)  7,786/  Both/  _/  Both | 51.4±0.1  >35 | FBS ≥ 126 mg/dL or self-reported diabetes medication use | 41.2  (36.4, 45.9) | 36.9  (32.2, 41.5) | 14.2  (11.3, 17.2) | 7 |
|  | Akter, 2014  ^95^ | Population based/  (2011)  7,541/  Both/  51.4/  Both | 51.4±0.07 | FBS ≥ 126 mg/dL or self-reported diabetes medication use | 44.0  (40.5, 47.5) | 39.5  (36.0, 42.9) | _ | 5 |
| Pakistan | P. Mahar 2010  ^96^ | Population based/  19,211/  both/  28.3/  urban | 30-90 | FBS ≥ 126 mg/dL  OGTT ≥ 200 mg/dL  previous diagnosis or oral antidiabetic treatment | 75.0  (72.9-77.1) | _ | _ | 7 |
|  | T. Afghani  2007  ^97^ | Population-based/  (1997-2001)/  53,684/  both/  rural | >40 | FBS ≥126 mg/dL  2-HPP > 200 mg/dL | 19.7  (18.6-20.9) | _ | _ | 8 |
|  | A. Shera  1995  ^98^ | Population-based/  1,035/  both/  20/  rural | ≥ 25 | 2-HPP > 200 mg/dL  WHO criteria | 36.5  (27.7-46.0) | _ | _ | 5 |
| South Africa | Adedokun  2019  ^99^ | Population based/  (2017)  403/  Both/  98.8/  Urban | 43.3±12.5 | FBS ≥ 126 mg/dL  or a self-reported history of diabetes or current use of anti-diabetic therapy | 68.2  (55.3, 79.4) | 47.6  (34.8, 60.5) | _ | 6 |
|  | Bailey, 2016  ^100^ | Population based/  (2010)/  12496/  both/  33.3/  both | 37 | RBG ≥ 200 mg/dL  control : 7.8% ≥ HbA1c | 87.3 (85.2-89.1) | 35.5 (32.7-38.2) | 13.7 (11.8-15.8) |  |
|  | Shen, 2016  ^12^ | Population based/  (2010)  1099/  Both/  _/  Urban | 35-74 | FBS ≥ 126 mg/dL | 65.5  (57.0, 73.3) | 38.7  (30.7, 47.3) | 16.2  (10.5, 23.3) | 6 |
|  | Erasmus, 2012  ^101^ | Population based/  (2008-2009)/  642/  both/  21.9/  urban | 50.9 ± 9.1 | FBS ≥ 126 mg/dL | 44.2  (36.8-51.7) | _ | _ | 7 |
| Jordan | Rana Dajani, 2012  ^102^ | Population based/  (2008 and 2009)  792/  Both/  35.2/  Urban | >18 | FBS ≥ 126 mg/dL | 76.9  (66.0, 85.7) | 57.7  (46.0, 68.1) | 35.9  (25.3, 47.6) | 5 |
|  | Ajlouni, 2008  ^103^ | Population based/  (2002)  1,121/  Both/  35.1/  Urban | 55.5±10.6 | FBS ≥ 126 mg/dL | 74.8  (68.2, 80.8) | 66.1  (59.0, 72.7) | 40.6  (32.0, 49.6) | 6 |
| Lebanon | Fahs, 2017  ^104^ | Population base /2015/  1000/  Both/  50.1/  Both | 54.84 ± 15.12 | RBS ≥ 200 mg/dL | 73.1 (78.4-70.3) | _ | _ | 6 |
| Israel | R. Dankner 2008  ^105^ | Elderly based/  623/  (data of a cohort study)  /both/  both | 70.6 ± 6.9 | FBS ≥126 mg/dL  or RBS ≥ 200 mg/dL | 59 (51.8-65.9) | _ | _ | 8 |
| Comoros | Ben Ali, 2019  ^106^ | Population-based/  (2017)  902/  Both/  40.1/  Urban | 39.5±11.6 | FBS ≥126 mg/dL | 64.5  (49.4, 77.8) | _ | _ | 5 |
| Luxembourg | Alkerwi, 2013  ^107^ | Population based/  (2007-2008)  1,432/  Both/  _/  Both | _ | Self-reporting of anti-diabetic medications and/or FBS ≥ 126 mg/dL | 68.1  (55.8, 78.8) | _ | _ | 6 |
| Myanmar | Aung, ^108^ | Population based (2004)/  4,448/  Both/  44.8/  Both | 25-74 | FBS ≥126 mg/dL  and/or self-reported as diagnosed by health professionals | 44.3  (39.2, 49.6) | 24.3  (20.0, 29.0) | 07.0  (4.9, 1.4) | 6 |
|  |  | Population based (2014)/  1,372/  Both/  49.6/  Both |  |  | 69.4  (62.9, 75.2) | 47.8  (39.3, 56.5) | 19.3  (13.1, 26.8) |  |
| Ghana | Bijlholt, 2018  ^109^ | Population based/  (2012-2015)  132/  Both/  34.1/  Urban | 52.9±2.0  25-70 | FBS ≥126 mg/dL, glucose-lowering medication use, or self-reporting of prior diagnosis of T2DM by a health care professional | 59.1  (50.2, 67.6) | 56.3  (47.5, 51.9) | 27.5  (20, 36) | 6 |
|  |  | Population based/  (2012-2015)  49/  Both/  29.4/  Rural | 54.5±3.5  25-70 |  | 51.0  (36.3, 65.6) | 37.3  (24.1, 64.8) | 63.4  (48.4, 79.8) |  |
| Guinea | Balde, 2017  ^110^ | Population base/  2009/  1100/  Both/  53.5/  Both | 47.3±8.8/ 35-64 | FBS ≥ 126 mg/dL | 44.0  (32.5, 55.9) | 28.0  (18.2, 39.5) | 12.0  (5.6, 21.5) | 10 |
| Zambia | Bailey, 2016  ^100^ | Population based/  (2010)  45,767/  both/  33.1/  both | 33.3 | RBS ≥ 200 mg/dL  control : 7.8% ≥ HbA1c | 65.4  (62.8-68.0) | 22.2  (20.0-24.6) | 6.5  (5.2-7.9) | 9 |
| Burkina Faso | Y. Sagna 2014  ^111^ | Population-based/  2011/  467/  both/  52.7/  urban | 21-74 39.1 ± 1.9 | FBS ≥126 mg/dL  previous diagnosis or oral antidiabetic treatment | 74.1 (60.9-84.7) |  |  | 6 |
| Netherland | Bijlholt, 2018  ^109^ | Population based/  (2012-2015)  164/  Both/  49.4/  Both | 52.2±2.0  25-70 | FBS ≥126 mg/dL, glucose-lowering medication use, or self-reporting of prior diagnosis of T2DM by a health care professional | 79.2  (72.2, 85.2) | 73.3  (66.0, 79.7) | 63.4  (55.5, 70.8) | 6 |
|  | Snijder, M.B  2017 ^112^ | Population based  (Dutch)/  (2011-2015)/  22,113/  Both/  43.9/  Both | 18-70 | FBS ≥126 mg/dL, if the participant was using a glucose-lowering medication, and/or if the participants self-reported to have been diagnosed with diabetes by a health care professional | 81.2  (79.5, 82.8) | 65.5  (63.5, 67.4) | 28.3  (26.4, 30.2) | 7 |
| Germany | Bijlholt, 2018  ^109^ | Population based/  (2012-2015)  70/  Both/  65.7/  Both | 51.1±3.0  25-70 | FBS ≥126 mg/dL, glucose-lowering medication use, or self-reporting of prior diagnosis by a health care professional | 72.5  (59.3, 81.8) | 72.9  (60.9, 82.8) | 62.7  (50, 74.2) | 6 |
|  | C. Heidemann 2016 ^113^ | Population-based/  (1997-1999)  6695/  both/  49.4/  both | 47.4 ± 0.5 (18-79) | HbA1C ≥ 6.5 % | 60.3  (56.3-64.1) | _ | _ | 9 |
|  |  | Population-based /  (2008-2011)/  7,017/  both/  49.8/  both | 45.4 ± 0.6 (18-79) |  | 78.2  (74.8-81.3) | _ | _ |  |
|  | A. Icks 2013  ^114^ | Population based/  (2006-2008)/  2,611/  both/  both | 40-82 | OGTT ≥ 200 mg/dL | 68.1  (62.9-73.0) | _ | _ | 6 |
|  | Meisinger, 2010,  ^115^ | Population-based/  (2006-2008)/  1,653/  Both/  45.8/  Both | 35-59 | FBS ≥126 mg/dL | 52.8  (40.6, 64.7) | _ | _ | 6 |
|  | H. Hauner 2008 ^116^ | Population-based/  2005/  35,869/  both/  48.9/  both | 51.7 ± 16.1 | FBS ≥126 mg/dL  or RBS ≥ 200 mg/dL | 92.3 (91.4-93.1) | _ | _ | 8 |
|  | W. Rathmann, 2003 ^117^ | Population-based/  (1999-2001)/  1,653/  Both/  51.8/  Both | 55-75 | FBS ≥126 mg/dL  or 2-HPP ≥ 200 mg/dL | 50.6  (44.2, 57.0) | _ | _ | 5 |
| England | Y.-T. Huang 2020 ^118^ | Elderly-based/  2004/  7,666/  both/  45/  both | 66.6 ± 9.9 | HbA1C ≥ 6.5% | 76.2  (73.1-79.2) | _ | _ | 9 |
|  |  | Elderly-based/  2012/  7,729/  both/  44.6/  both | 67.6 ± 9.5 |  | 77.2  (74.7-79.6) | _ | _ |  |
|  | E. S. M. dos Santos 2020  ^119^ | Elderly based/  5301/  (data of a cohort study)/  both/  both | 67 | HbA1C ≥ 6.5% | 76.2  (72.8-79.4) | _ | _ | 7 |
|  | Bijlholt, 2018  ^109^ | Population based/  (2012-2015)  70/  Both/  41.2/  Both | 54.6±2.5  25-70 | FBS ≥126 mg/dL, glucose-lowering medication use, or self-reporting of prior diagnosis by a health care professional | 72.6  (62.8, 80.8) | 66.7  (56.6, 75.7) | 40.0  (29.8, 50.9) | 6 |
|  | Alison Moody, 2016  ^120^ | Population-based/  (2009-2013)/  18,399/  Both  45.0/  Both | 51.3±0.1  >16 | Those who reported that their doctor had diagnosed them with diabetes (except only when pregnant), as well as those taking medication prescribed only for diabetes, were categorized as ‘doctor-diagnosed diabetes’. Those not diagnosed with diabetes were categorized as ‘undiagnosed diabetes’ if HbA1c ≥48 mmol/mol (>6.5%), IGR if 42–47 mmol/mol (6.0–6.5%) and normoglycaemic if <42 mmol/mol (<6.0%) | 77.5  (75.3, 79.6) | _ | _ | 6 |
|  | Thomas, 2005  ^121^ | Elderly-based/  (1998-2000)/  4,252/  Men/  Urban | 60-79 | FBS ≥126 mg/dL | 60.0  (55.6, 64.2) | _ | _ | 8 |
|  |  | Elderly-based/  (1999-2001)/  4,286/  Female/  Urban |  |  | 54.0  (48.7, 58.6) | _ | _ |  |
|  | L. Riste 2001  ^122^ | Population-based/  1,063/  both/  48.6/  both | 25-79 | FBS ≥ 126 mg/dL or RBS > 200 mg/dL for all other samples of whole blood | 54.2  (47.1-61.3) | _ | _ | 6 |
|  | D simmons, 1991  ^123^ | Population-based/  (1986-1989)/  7,221/  Both/  -/  Both | >20 | OGTT ≥ 200 mg/dL | 66.2  (61.8, 70.4) | _ | _ | 6 |
|  | D Simmons, 1989  ^124^ | Population-based/  (1987)/  4,673/  Both/  -/  Both | 20-80 | OGTT ≥ 200 mg/dL | 70.8  (64.3, 76.7) | _ | _ | 6 |
| France | Lailler, 2020  ^125^ | Population-based/  (2014-2016)/  2,270/  47.4 | 47.3  18-74 | FBS ≥126 mg/dL  for undiagnosed diabetes | 76.8  (69.7, 82.9) | 78.2  (70.2, 85.1) | _ | 7 |
|  | Carrère, 2017 ^126^ | Population based/  (2014)  2,252/  Both/  43.5/  Both | 43.55  18-74 | Use of oral or injectable anti-diabetic treatment, or no treatment but an FBS ≥126 mg/dL  or more and HbA1c ≥ 6.5% | 78.0  (71.6, 85.1) | 76.5  (69.0, 82.9) | 16.3  (10.9, 23.2) | 6 |
|  | S. Fuentes 2018  ^127^ | Population based/  2013/  16,340/  both/  both | 18-70 | FBS ≥ 126 mg/dL | 67.2  (64.1-70.2) | _ | _ | 7 |
|  | C. Bonaldi, 2006  ^128^ | Population based/  (2012)/  2006/  both/  both | 18-74 | FBS ≥ 126 mg/dL or HbA1c ≥ 6.5% | 70.2  (60.4-78.8) | _ | _ | 6 |
| Italy | Omboni, 2013  ^129^ | Population based/  (2007)  344/  Both/  47.4/  Both | 54.9 ± 15.1 19–85 | FBS ≥ 126 mg/dL | 57.1  (37.2, 75.5) | 35.7  (18.6, 55.9) | 32.1  (15.8, 52.3) | 5 |
|  | Scuteri, 2009, ^130^ | Population based/  6,123/  Both/  42.5/  Both | 14-102 | FBS ≥ 126 mg/dL after 9 and <24 h fasting | 66.7  (60.0, 71.9) | 43.4  (36.5, 50.8) | 28.7  (22.5, 35.6) | 5 |
|  | Muntoni, 2009  ^131^ | Population-based/  (2002-2005)/  4,737/  Both/ | >20 | FBS ≥ 126 mg/dL and or gistory of diabetes | 60.6  (57.0, 64.3) | _ | _ | 5 |
|  | Gnasso, A, 1997  ^132^ | Population based/  (1994)  742/  Both/  58.6/  Urban | >45 | FBS > 140 mg/dl and/or use of ant diabetic agents | 76.7  (65.3, 85.8) | 64.4  (52.3, 75.2) | 19.2  (10.9, 30) | 5 |
| Switzerland | Kaiser, A  2012  ^133^ | Population based/  (2003)  6,181/  Both/  47.5/  Both | 35-75 | FBS ≥ 126 mg/dL and/or oral hypoglycemic treatment and/or insulin | 65.3  (60.4, 70.0) | 56.4  (51.3, 61.3) | _ | 6 |
| Sweden | L. Bennet 2011  ^134^ | 175/  both/  54.28/  urban |  | FBS ≥ 126 mg/dL  OGTT ≥ 200 mg/dL  previous diagnosis | 66.7  (49.0-81.4) | _ | _ | 4 |
| Finland | H. Ylihärsilä, 2005  ^135^ | Population-based/  (1992)/  2087/  Both/  45.8/  Both | 45-64 | FBS ≥ 126 mg/dL , 2-HPP ≥ 200 mg/dL | 56.0  (48.5, 63.4) | _ | _ | 4 |
|  | L. Hiltunen 1994  ^136^ | Elderly-based/  379/  1991/  both/  37.2/  both | ≥ 70 | 2-HPP ≥ 200 mg/dL | 25.8  (21.5-30.6) | _ | _ | 7 |
| Malta | S. Cuschieri 2020  ^137^ | Population based/  (2014-2016)  3947/  both/  50.6/  both | 47 (18-70) | FBS ≥ 126 mg/dL | 61.2  (56.2-65.9) | _ | _ | 8 |
| Norway | Kristian Midthjell, 1995  ^138^ | Population-based/  (1984-1986)/  76,855/  Both/  49.0/  Both | >20 | FBS ≥ 126 mg/dL and,or 2-HPP ≥ 200 mg/dL | 85.4  (84.0, 86.7) | _ | _ | 5 |
| Ireland | Jennifer M. O Connor, 2013  ^139^ | Population-based/  (2010-2011)/  1,873/  Both/  47.6/  Both | 50-69 | HbA1c > 6.5% | 58.6  (50.9, 66.0) | _ | _ | 5 |
| Denmark | Moustgaard, 2005  ^140^ | Population based/  (1999-2001)/  256/  Both/  Both | 46 | FBS ≥ 126 mg/dL and/or 2-HPP ≥ 200 mg/dL were taken to indicate diabetes | 38.5  (19.8, 57.2) | _ | _ | 5 |
| Poland | Monika Puzianowska-Kuznicka, 2021  ^141^ | Elderly-based/  4,089/  Both/  52.0/  Both | >65 | FBS > 125 mg/dL | 81.7  (79.0, 84.2) | _ | _ | 6 |
| Greenland | Marit Rgensen  2002  ^142^ | Population based/  (1999-2001)/  894/  Both/  Both | 49  35–86 | FBS ≥ 126 mg/dL and/or 2-HPP ≥ 200 mg/dL were taken to indicate | 29.9  (20.5, 40.6) | _ | _ | 5 |
| Greece | N. Papazoglou, 1995  ^143^ | Elderly-based/  (1991-1992)/  647/  Both/  46.7/  Urban | >65 | Two abnormal glucose values according to the WHO criteria | 68.8  (61.6, 75.4) | 30.7  (24.2, 37.8) | _ | 4 |
| Spain | A. Aguayo 2016,  ^144^ | Population based/  (2010-2012)  828/  both/  44.5/  both | ≥18 | - | 56.1  (44.7-67.0) | _ | _ | 7 |
|  | Ana Redondo  2011  ^145^ | Population based (1995)/  1,480/  Both  47.9/  Both | 54.5±11.4 | FBS ≥ 126 mg/dL or those patients receiving treatment  with glucose-lowering drugs | 67.4  (59.0, 75.0) | 48.2  (39.7, 56.8) | 6.4  (3.0, 11.8) | 6 |
|  |  | Population based (2000)/  2,539/  Both/  48.9/  Both | 53.7±11.0 |  | 58.4  (52.3, 64.4) | 42.3  (36.3, 48.5) | 4.1  (2.0, 7.2) |  |
|  |  | Population based (2005)/  5,627/  Both/  47.1/  Both | 54.3±11.0 |  | 74.6  (70.8, 78.2) | 54.9  (50.7, 59.1) | 13.9  (11.1, 17.0) |  |
| USA | Kazumi Tsuchiya, 2021  ^146^ | Population-based/  (2011-2016)/  15,201/  Both/  47.9/  Both | 47.5±0.4 | HbA1c levels ≥ 6.5% | 83.3  (81.5, 85.0) | _ | _ | 8 |
|  | Rajat Kalra,  2021  ^147^ | Population based/  4,861/  (2005-2008)/  Both/  Both | 18-44 | FBS ≥126 mg/dL after ≥8 hours of fasting, RBS ≥200 mg/dL, plasma HbA1C (HbA1c) ≥ 6.5%, self-reported history of diabetes mellitus, or current use of oral hypoglycemic or insulin | 74.1  (66.7, 81.5) | 29.8  (21.3, 38.3) | 6.7  (1.5, 11.8) | 8 |
|  |  | Population based/  5,235  (2009-2012)/  Both/  Both |  |  | 61.1  (50.9, 71.3) | 49.0  (37.4, 60.5) | 14.0  (8.8, 19.2) |  |
|  |  | Population based/  5,075  (2013-2016)/  Both/  _/  Both |  |  | 71.3  (64.0, 78.4) | 55.9  (48.3, 63.5) | 17.4  (11.6, 23.3) |  |
|  | Jaewon Lee, 2021  ^148^ | Population based/  (2010-2012)  14,328/  Both/  Both | >20 | FBS ≥ 126 mg/dL, HbA1c ≥6.5% (48 mmol/mol), post 2-hour plasma glucose after 75 g oral glucose  ≥200 mg/dL, self-report of previous diagnosis of diabetes by medical professionals, or taking glucose-lowering agents or insulin (survey or  prescription medication check) | 71.3  (69.2, 73.3) | 56.5  (54.3, 58.8) | 29.0  (27.0, 31.1) | 8 |
|  | Shisi He, 2020  ^149^ | Population based/  5,337  (2010-2018)/  Both/  Both | 43.7±16.8 | FBS ≥ 126 mg/dL or HbA1c ≥ 6.5% or taking glucose lowering medication | 63.6  (55.7, 70.4) | 45.8  (38.3, 53.4) | 40.0  (22.6, 16.7) | 6 |
|  |  | Population based/  2,571  (2013-2014)/  Both/  Both | 40.0±19.3 |  | 71.7  (66.6, 76.4) | 60.8  (55.3, 66.0) | 33.6  (28.6, 38.4) |  |
|  | Y. J. Cheng 2019  ^150^ | Population-based/  (2011-2016)/  7575/  both/  48%/  both | 47.5 ( ≥ 20) | FBS ≥ 126 mg/dL or HbA1c ≥ 6.5% or 2-HPP ≥ 200 mg/dL | 85.7 (84.3-87.1) | _ | _ | 8 |
|  | Emily M. Bucholz, 2018  ^151^ | Population based/  (2011-2014)  11,083/  Both/  Both | 18-39 | FBS ≥126 mg/dL or HbA1c  ≥ 6.5%, or self-reported use of insulin or oral agents | 70.0  (64.5, 75.1) | _ | _ | 7 |
|  | S. P. Fisher-Hoch, 2015  ^152^ | Population-based/  (2004-2014)/  2,838/  both/  43.4/  urban | 46 ± 0.68 | HbA1C ≥ 6.5, self-report, previous diagnosis, use of diabetes medication | 56.5  (52.9-60.0) | _ | _ | 8 |
|  | Redondo A, 2011, ^153^ | Population based/  (1995)/  1,480/  Both/  47.9/  Both | 54.5±11.4 | FBS >126 mg/dL or those patients receiving treatment  with glucose-lowering drugs | 67.4  (59.0, 75.0) | 48.2  (39.7, 56.8) | 6.4  (3.0, 11.8) | 8 |
|  |  | Population based/  (2000)  2,539/  Both/  48.9/  Both | 53.7±11.0 |  | 58.4  (52.3, 64.4) | 42.8  (36.3, 48.5) | 4.1  (2.0, 7.2) |  |
|  |  | Population based/  (2005)  5,627/  Both/  47.1/  Both | 54.3±11.0 |  | 74.6  (70.8, 78.2) | 54.9  (50.7, 59.1) | 13.9  (11.1, 17.0) |  |
|  | Parton JM,  2011,  ^154^ | Population based/  (2003-2007)/  14,374/  Both/  Both | >45 | FBS ≥ 126 mg/dL or a RBS ≥ 200 mg/dL, or must  have been medicated with pills or insulin | 88.4  (87.1, 89.6) | 83.8  (82.3, 85.2) | 44.6  (42.7, 46.5) |  |
|  | C. C. Cowie, 2010 ^155^ | Population based/  (2003-2006)  9025/  both/  both | ≥ 20 | HbA1C ≥ 6.5% | 81.3  (78.5-83.8) | _ | _ | 6 |
|  | C. C. Cowie 2009 ^156^ | Population-based/  (2005-2006)  7267/  both/  both | ≥ 20 | RBS ≥ 200 mg/dL | 76.2  (73.3-78.9) | _ | _ | 7 |
|  | Lorna e. Thorpe, 2009  ^157^ | Population-based/  (2004)/  1,336/  Both/  41.9  Urban | >20 | FBS ≥ 126 mg/dL | 70.0  (62.5, 76.9) | _ | _ | 7 |
|  | Hertz 2006 ^158^ | Population based (Mexican American)/  (1999-2002)  1,010/  Both/  52.5/  Both | >20 | FBS ≥ 126 mg/ dL, by a physician, use of anti diabetic medications | 77.4  (69.6, 84.3) | 62.8  (53.0, 74.4) | 17.9  (11.8, 25.5) | 5 |
|  |  | Population based (White, non-Hispanic)/  (1999-2002)  2,037/  Both/  48.4/  Both |  |  | 64.5  (56.4, 72.0) | 47.1  (37.0, 57.2) | 24.5  (18.0., 32.0) |  |
|  | Menke, 2015,  ^159^ | Population-based/  (1988-1994)/  8,478/  Both/  Both | >20 | HbA1c ≥6.5% or FBS ≥ 126 mg/dL or greater, 2-HPP ≥ 200 mg/dL | 68.5  (65.0, 71.8) | _ | _ | 8 |
|  |  | Population-based/  (1999-2000)/  2,168/  Both/  Both |  |  | 76.5  (69.8, 82.3) | _ | _ |  |
|  |  | Population-based/  (2001-2002)/  2,479/  Both/  Both |  |  | 72.2  (66.4, 77.4) | _ | _ |  |
|  |  | Population-based/  (2003-2004)/  2.299/  Both/  Both |  |  | 73.2  (67.4, 78.6) | _ | _ |  |
|  |  | Population-based/  (2005-2006)/  2,191/  Both/  Both |  |  | 74.3  (68.2, 79.7) | ­_ | _ |  |
|  |  | Population-based/  (2007-2008)/  2,901/  Both/  Both |  |  | 71.1  (66.1, 75.7) | _ | _ |  |
|  |  | Population-based/  (2009-2010)/  3,118/  Both/  Both |  |  | 71.2  (66.3, 75.7) | _ | _ |  |
|  |  | Population-based/  (2011-2012)/  2,781/  Both/  Both |  |  | 74.8  (69.9, 79.3) | _ | _ |  |
|  | Schneiderman, N, 2014, ^160^ | Population based/  (2008-2011)  16,385/  Both/  40.0/  Urban | 18-74 | FBS ≥ 126 mg/dL, a 2-HPP >200 mg/dl (11.2 mmol/l), HbA1c ≥ 6.5% (48mmol/mol), or documented use of hypoglycemic agents(scanned medications) | 58.7  (56.8, 60.5) | _ | _ | 7 |
|  | Sims, M, 2011  ^13^ | Population based/  (2000-2004)  4,303/  36.7/  Both | 21.94 | FBS ≥ 126 mg/dl, or confirmed medication usage from the medication inventory, or self-reported use of ant diabetic medications within the past 2 weeks of the examination, or self-reported diabetes diagnosis | 89.2  (86.9, 91.7) | 86.0  (83.4, 88.4) | 32.6  (31.2, 34.0) | 6 |
|  | McDonald, 2009  ^161^ | Elderly based/  (1999-2004)  3,810/  Both/  49.6/  Both | >65 | FBS ≥ 126 mg/dl, or they reported being told by a physician that they had diabetes or they were taking glucose-lowering medication | 71.4  (68.2, 74.5) | 50.9  (47.4, 54.3) | 25.5  (22.6, 28.6) | 5 |
|  | L. A. Jaber 2003 ^162^ | Population based/  542/  both/  39.4/  urban | ≥ 20 | FPG ≥ 126 mg/dL  OGTT ≥ 200 mg/dL | 50.0  (40.1-59.9) | _ | _ | 6 |
| Canada | Hosseini,  2019, ^163^ | Population based/  (2007-2011)  6,807/  Both/ | 20–79 | individuals with diagnosed diabetes that had been confirmed by health professionals | 62.5  (58.2, 66.8) | - | - | 7 |
|  | Laura C. Rosella, 2015  ^164^ | Population-based/  (2007-2011)/  3,494/  Both/  /  Both | >20 | FBS ≥ 126 mg/dL,HbA1c ≥ 6.5% (≥48 mmol/mol) | 70.7  (64.3, 76.6) | _ | _ | 7 |
|  | Leiter, 2001,  ^165^ | Population-based/  (1996)/  9,042/  Both/  48.1/  Both | >40 | FBS ≥ 126 mg/dL | 88.0  (86.3, 89.5) | _ | _ | 6 |
|  | T. Kue Young, 2001  ^166^ | Population-based/  (1989-1990)/  2,150/  Both/  -/  Both | 18-74 | FBS ≥ 126 mg/dL | 67.4  (59.0, 74.9) | _ | _ | 4 |
|  | Aaron Leong,  2013,  ^167^ | Population-based/  (2009)/  1,598/  Both/ | 49.7±16.4 | FBS ≥ 126 mg/dL | 60.3  (52.3, 67.6) | _ | _ | 6 |
| Laos | Vonglokham, M, 2019  ^168^ | Population based/  (2013)  2,492/  Both/  40.3/  Both | 38.9±12.3  18-64 | FBS ≥ 126 mg/dL, or using insulin or oral hypoglycemic drugs, or having a history of diagnosis of diabetes | 58.1  (47.4, 64.2) | 40.3  (29.1, 45.5) | 10.9  (5.4, 15.8) | 6 |
| Tanzania | Stanifer, J, W  ^169^ | Population based/  (2014 and 2015)  481/  Both/  25.6/  Both | >18 | HbA1c level ≥ 7 or current known use of anti-hyperglycemic medications to treat diabetes | 35.6  (21.9, 51.2) | 33.3  (20.0, 48.9) | _ | 6 |
|  | Ruhembe, C.C, 2014  ^170^ | Population based/  (NR)  640/  Both/  45/  Urban | 43.6±11.3  >30 | FBS ≥ 126 mg/dL | 49.2  (45.2, 53.2) | _ | _ | 5 |
| Mozambique | Silva-Matos, C, 2011  ^171^ | Population based/  (2005)  2,343/  Both/  40.2/  Both | 25-64 | Fasting capillary glucose ≥ 110 mg/dL or treatment with insulin and/or oral blood glucose-lowering drugs | 13.3  (0.0, 27.0) | _ | _ | 5 |
| Afghanistan | K. M. Islam Saeed, 2017  ^172^ | Population-based/  1,129/  both/  47.4/  urban | (25-79) 41.7 ± 13.1 | FBS ≥ 126 mg/dL | 33.0  (24.4-42.6) | _ | _ | 8 |
| Turkey | Selcuk, K.T 2015 ^173^ | Population based/  (2007 and 2008)  12,915/  Both/  33.4/  Both | 52.5±12.9  >30 | FBS ≥ 126 mg/dL or treatment with insulin and/or oral blood glucose-lowering drugs | 87.7  (86.0, 89.2) | _ | _ | 6 |
|  | Ilhan satman, 2002  ^174^ | Population-based/  (1997-1998)/  24,788/  Both/  44.7/  Both | ≥ 20 | 2-HPP ≥ 200 mg/dL | 68.0  (65.8, 70.2) | _ | _ | 8 |
| Syria | N. Albache 2010  ^175^ | Population-based/  2006/  1,168/  both/  47.7  /urban | 44.7 ± 12.7 ≥ 25 | FBS ≥126 mg/dL  HbA1C ≥ 6.5 | 73.3  (66.6, 79.2) | _ | _ | 6 |
| Saudi Arabia | Bahijri 2016  ^176^ | Population-based/  1,420/  both/  47/  urban | 36 ± 15.4 ≥ 18 | FBS ≥ 126 mg/dL  HbA1C ≥ 6.5% | 91.5  (87.0-94.8) | _ | _ | 7 |
|  | K. Al‐Rubeaan 2015  ^177^ | Population-based/  2006/  18,034/  both/  both | ≥ 30 | FBS ≥ 126 mg/dL | 59.7  (58.2-61.1) | _ | _ | 7 |
|  | K. Al-Rubeaan 2014  ^178^ | Population-based/  (2007-2009)/  53,370/  both/  51.5/  both | All ages | FBS ≥ 126 mg/dL | 44.5  (43.3-45.8) | _ | _ | 10 |
|  | C. El Bcheraoui 2013  ^179^ | Population-based/  both/  both | ≥ 15 | HbA1C ≥ 6.5% | 73.8  (71.5-76.1) | _ | _ | 6 |
|  | N. A. Al-Baghli 2010  ^180^ | Population-based/  (2004-2005)/  196,769/  both/  50.5/  both | ≥ 30 | FBS ≥ 126 mg/dL  RBS ≥ 200 mg/dL | 90.9  (90.6-91.2) | _ | _ | 7 |
|  | M. M. Al-Nozha 2004  ^181^ | Population-based/  16,917/  both/  47/  both | 30-70 | 1997 American Diabetes Association (ADA) criteria | 72.0  (70.6-73.4) | _ | _ | 7 |
| Oman | J. Al‐Lawati 2002  ^182^ | Population-based/  2000/  5,838/  both/  50.2/  both | ≥ 20 | FBS ≥ 126 mg/dL | 33.4  (29.5-37.4) | _ | _ | 7 |
|  | A. Al Riyami 2012  ^183^ | Population-based/  2007  3,370/  both/  46.8/  both | ≥ 18 | FBS ≥ 126 mg/dL  OGTT ≥ 200 mg/dL | 47.9  (43.0-52.9) | _ | 16.7  (13.2-20.7) | 9 |
| Malaysia | H. Ismail 2018  ^184^ | Population based/  19,935/  both/  47.6/  urban | ≥ 18 | FBS ≥ 126 mg/dL | 39.7  (38.1-41.4) | _ | _ | 7 |
|  | Steven T. YEN, 2017  ^185^ | Population based/  (2005 and 2006)  2,063/  Both/  42.3/  Both | 25-65 | FBS ≥ 126 mg/dL | 6.5  (5.5, 7.7) | 4.9  (4.0, 5.9) | _ | 6 |
|  | Ho BK, 2011  ^186^ | Elderly based/  (2011)  2,708/  Both/  46.5/  Both | >60 | Self-report by a respondent who  had been told by a doctor or medical assistant that they have diabetes | 65.2  (60.3, 69.9) | 57.1  (53.8, 60.3) | 12.4  (10.4, 14.7) | 7 |
|  | Rampal, S, 2010, ^187^ | Population based/  (2004)  7,683/  Both/  39.6/  Both | >30 | FBS ≥ 126 mg/dL or a self-reported diabetic on treatment | 45.0  (41.4, 48.7) | 42.7  (39.1, 46.3) | 25.1  (20.7, 30.1) | 6 |
| Indonesia | Laurentius A. Pramono, 2010  ^188^ | Population-based/  (2007)/  18,956/  Both/  46.4/  Both | >18 | OGTT > 200 mg/dL no history of diagnosis and treatment of diabetes | 26.5  (23.8, 29.2) | _ | _ | 6 |
| Portugal | Barreto, M, 2018  ^189^ | Population based/  (2015)  4,756/  Both/  48.2/  Both | 25-74 | A Self-reported diagnosis of diabetes performed by a medical doctor. Self-reported use of anti-diabetic medication prescribed by a medical doctor or HbA1c ≥ 6.5 (47.5mmol/mol) Control: FBS < 126 mg/dL | 87.1  (81.2, 91.3) | 79.7  (73.4, 84.8) | 63.2  (57.1, 96.0) | 5 |
|  | L. Gardete-Correia 2010  ^190^ | Population-based/  51,67/  2001/  both/  both | 20-79 | 2-HPP,OGTT ≥ 200 mg/dL, FBS ≥ 126 mg/dL | 56.4  (52.3-60.4) | _ | _ | 7 |
| Russia | Bikbov, M.M  2019,  ^191^ | Population based/  (2015-2017)  5,899/  Both/  43.8/  Both | 59.0±10.7  >40 | FBS ≥ 126 mg/dL or by a self-reported history of physician diagnosis of diabetes mellitus  or by a history of drug treatment for diabetes | 72.8  (69.0, 76.0) | 59.1  (55.4, 62.8) | 58.5  (53.7, 63.3) | 5 |
|  | Dedov, 2016  ^192^ | Population-based/  (2013-2015)  26620/  both/  51.5/  both | 43.2 ± 15.4 | HbA1C ≥ 6.5% | 46.1 (43.5-48.7) | _ | _ | 9 |
| Jamaica | Cunningham-Myrie, C, 2013,  ^193^ | Population based/  (2007 and 2008)  2,848/  Both/  31.3/  Both | 15-74 | FBS ≥ 126 mg/dL , or if participant reported being on medication for DM | 76.1  (70.3, 81.8) | 71.1  (64.7, 76.9) | 30.6  (24.7, 37.1) | 5 |
| Seychelles | Faeh, D, 2007, ^194^ | Population based/  (2004)  1,255/  Both/  45.2/  Both | 25-64 | FBS ≥ 126 mg/dL, 2-HPP ≥ 200 mg/dL or current history of ant diabetic medication | 54.0  (46.0, 62.0) | _ | _ | 5 |
| Nepal | Gyawali, B, 2018, ^195^ | Population based/  (2016 and 2017)  2,310/  Both/  32.0/  Urban | 47.3±9.9  >25 | Participants had previously been diagnosed by a physician and/or were on anti diabetic medication and/or had FBS ≥ 126 mg/dL | 65.0  (58.7, 76.6) | 60.5  (54.4, 66.4) | 13.6  (9.8, 18.3) | 6 |
|  | M. Chhetri 2009  ^196^ | Elderly-based/  1633/  both/  both | ≥ 60 | FBS ≥ 126 mg/dL | 33.2 (28.7-37.9) | _ | _ | 6 |
| Thailand | Wichai, 2011  ^197^ | Population based/  (2009)  18629/  both/  48.1/  both | ≥20 | FBS ≥ 126 mg/dL | 72.5 (70.6-74.5) | _ | _ | 6 |
|  | Porapakham, Y, 2008,  ^198^ | Elderly based/  (2004)  19,374/  Both/  48.5/  Both | >60 | FBS ≥ 126 mg/dL or use of medication for the treatment of diabetes during the previous 2 weeks | 58.8  (57.0, 60.5) | 53.1  (50.7, 55.4) | 26.4  (23.6, 29.3) | 7 |
|  | Wichai, 2007  ^199^ | Population based/  (2004)  37138/  both/  both | ≥15 | FBS ≥ 126 mg/dL | 46.0 (44.0-48.0) | _ | _ | 6 |
|  | Wichai, 2003  ^200^ | Population based/  (2000)  5350/  both/  both | ≥35 | FBS ≥ 126 mg/dL | 50 (45.5-54.4) | _ | _ | 6 |
| Taiwan | Pan, W.H, 2003,  ^201^ | Population based/  (1993-1996)  2,691/  Both/  _/  Both | ≥19 | A person was diagnosed with diabetes by a physician and was on medication for diabetes mellitus, or an FBS ≥ 110 mg/dL | 65.0  (56.3, 72.9) | _ | _ | 4 |
| Kazakhstan | Supiyev,A  ^202^ | Elderly based/  (2012-2015)  953/  Both/  44.2/  Both | 60.7±7.3  50-75 | FBS ≥ 126mg/dL and/or being on diabetes medication | 72.3  (64.1, 80.4) | 65.6  (56.9, 74.2) | 27.7  (19.6, 35.9) | 5 |
| Nigeria | Makusidi, M.A, 2013  ^203^ | Population based/  (NR)  535/  Both/  62.1/  Both | 37±17  15-80 | RBS > 200mg/dL | 11.4  (3.5, 28.9) | _ | _ | 5 |
| Tunisia | H. B. Romdhane 2014  ^204^ | Population-based/  7700/  both/  41.9/  both | ≥ 35 | FBS ≥ 110 mg/dL  previous diagnosis or oral antidiabetic treatment | 52.1  (50.0-54.2) | 28.7  (26.8-30.6) | _ | 9 |
|  | R. Bouguerra 2007  ^205^ | Population-based/  (1996-1997)  37,700/  both/  34.8/  both | ≥ 20 | FBS ≥ 126 mg/dL | 25.0  (23.6-26.4) | _ | _ | 8 |
| Tonga | S. Colagiuri 2002  ^206^ | Population based/  (1998-2000)/  1,024/  both/  42.3/  both | 41.3 ± 14.3 | OGTT ≥ 200 and elevated HbA1C or FBS ≥ 126 mg/dL | 31.0 (23.8-39.0) | _ | _ | 3 |
| Kenya | Mohamed, S.F, 2018  ^207^ | Population based/  (2015)  4,069/  Both/  _/  Both | 18-69 | FBS ≥ 126 mg/dL or a self-report of a previous diagnosis of diabetes by a health care professional or currently receiving treatment for diabetes | 43.7  (29.1, 59.5) | 21.3  (12.0, 35.1) | 7.0  (3.6, 13.2) | 7 |
|  | Oti, S.O  2013  ^208^ | Population based/  (2008-2009)/  5,190/  Both/  _/  Both | >18 | RBS ≥ 11.1 mM or previously diagnosed by a health professional or confirmed by oral glucose tolerance test in accordance with WHO criteria | 33.9  (28.5, 39.6) | 24.2  (19.4, 29.4) | 7.0  (4.4, 10.6) | 6 |
| Singapore | Huang , O.S,  2009  ^209^ | Population based/  (2004 and 2006)  3,280/  Both/  _/  Both | 40-80 | RBS ≥ 11 mmol/l, use of diabetic medication or a previous physician diagnosis | 86.8  (84.2, 89.1) | _ | _ | 5 |
|  | Ryan Man, 2019  ^210^ | Population based/  (2004-2011)  6,904/  Both/  47.7/  Both | 61.1±9.9  40-80 | RBS ≥ 200 mg/dL and/or HbA1c > 6.5% or self-reported the use of diabetes medication | 69.3  (67.4, 71.1) | _ | _ | 5 |
| Angola | Pedro, J.M  2018  ^11^ | Population based/  (2013 and 2014)  2,348/  Both/  48.0/  Both | 32.5±13.6  15-64 | FBS ≥ 126mg/dL and/or use of ant diabetic drugs during the previous 2 weeks | 10.8  (7.3, 15.5) | 4.5  (2.1, 8.0) | 2.7  (1.0, 5.8) | 5 |
| Cameron | Katte, J.C, 2014  ^211^ | Population based/  (2012)  1,702/  Both/  43.2/  Urban | 45.7±14.0  >18 | Diagnosed by medical doctors or other health professionals and were on drug treatment in the past two weeks | 34.2  (23.5, 46.3) | 24.4  (17.6, 39.1) | 19.  (10.9, 30.1) | 6 |
| Sudan | M. A. Eltom 2018  ^212^ | Population-based/  2015/  5,242/  both/  39.2/  both | 43.2 ± 16.0 ≥ 18 | FBS ≥ 126 mg/dL  RBS ≥ 200 mg/dL | 70.9  (68.0-73.8) | _ | _ | 8 |
|  | W. M. Elmadhoun 2016  ^213^ | Population-based/  2015/  954/  both/  45.7/  urban | 18-90 39.5 ± 16.7 | FBS ≥ 126 mg/dL  RBS ≥ 200 mg/dL | 68.7  (61.4-75.3) | _ | _ | 7 |
| Benin | F. Djrolo 2015  ^214^ | Population-based/  4,597/  2008/  both/  42.1/  both | 37.2 ± 16.5 ≥ 18 | FBS ≥ 126 mg/dL | 12.8  (9.7-16.4) | _ | _ | 7 |
| Uganda | Bahendeka, 2016  ^215^ | Population-based/  (2014)/  3,689/  Both/  39.8/  Both | 18-69 | FBS ≥ 126 mg/dL currently on medication for diabetes mellitus | 50.0  (35.0, 65.2) | _ | _ | 7 |
| Mongolia | Dugee Otgontuya, 2012  ^216^ | Population-based/  (2009)/  1,470/  Both/  50.3/  Both | 15-64 | FBS ≥ 110 mg/dL  mg/dl) | 62.9  (55.2, 70.0) | 46.9  (39.3, 54.5) | 16  (10.9, 22.3) | 6 |
| Cambodia | Dugee Otgontuya, 2012  ^216^ | Population-based/  (2010)/  5,124/  Both/  48.6/  Both | 25-64 | FBS ≥ 110 mg/dL | 53.8  (46.8, 60.8) | 48.1  (41.1, 55.1) | 24.0  (18.4, 30.4) | 6 |
| Ethiopia | N. Dereje 2020  ^217^ | Population-based/  2017  627/  /both/  58.5/  both | ≥ 18 | FBS ≥ 126 mg/dL | 63.9  (46.2-79.2) | _ | _ | 6 |
|  | T. Endris 2019  ^218^ | Population-based/  587/  2019/  both/  39.7  /urban | 44.17 ± 13.36 ≥ 18 | FBS ≥ 126 mg/dL | 27.5  (14.6-43.9) | _ | _ | 7 |
|  | Bantie, 2019  ^219^ | Population based/  (2010)/  607/  both/  30/  urban | 35.2 ± 13.8 | FBS ≥ 126 mg/dL | 10.2 (7.92-12.9) | _ | _ | 5 |
|  | Aynalem,  2018,  ^220^ | Population-based/  (2016)/  402/  Both/  37.6  Urban | 31±6.5  15-78 | FBS ≥ 126 mg/dL and the American Diabetes Association diabetes mellitus classification criteria | 11.5  (2.4, 30.1) | _ | _ | 6 |
|  | Animaw,  2017,  ^221^ | Population-based/  (2015)/  1,405/  Both/  43.3/  Both | >18 | FBS ≥ 126 mg/dL | 23.9  (12.6, 38.7) | _ | _ | 6 |
|  | W. Seifu 2015  ^222^ | Population-based/  (2008-2009)/  4,371/  both/  47.9/  both | 15-64 40.45 ± 14.94 | FBS ≥126 mg/dL previous diagnosis or oral antidiabetic treatment | 34.8  (23-7-47.2) | _ | _ | 7 |
|  | S. M. Abebe 2014  ^223^ | Population based/  2,141/  both/  48/  both | ≥35 | FBS ≥ 126 mg/dL | 31.0  (21.0-42.0) | _ | _ | 6 |
| Southern Cone of Latin America | Irazola, V  2017  ^224^ | Population based/  (NR)  7,441/  Both/  41.7/  Urban | 35-75 | FBS ≥ 126 mg/dL or self-reported history of diabetes, or previous diagnosis of diabetes by a health professional | 79.8  (77.3, 82.2) | 58.8  (55.8, 61.8) | 28.9  (26.2, 31.8) | 5 |
|  | Shen, 2016  ^12^ | Population based/  (2010)  7,355/  Both/  _/  Urban | 35-74 | FBS ≥ 126 mg/dL | 81.2  (78.6, 83.5) | 63.3  (60.3, 66.2) | 28.4  (25.7, 31.3) | 6 |
| South Asia | Shen, 2016  ^12^ | Population based/  (2010)  11,907/  Both/  _/  Urban | >20 | FBS ≥ 126 mg/dL | 81.7  (80.0, 83.3) | 64.5  (62.5, 66.5) | 29.0  (27.2, 31.0) | 6 |
| Tajikistan | Matthys, B,  2015  ^225^ | Population-based/  (2012)/  584  Both/  33.0/  Both | >45 | FBS ≥126 mg/dL or 2-H PP ≥ 11.1 mmol/l (200 mg/dl) | 49.2  (40.0, 58.3) | _ | _ | 4 |
| Peru | Shen, 2016  ^12^ | Population based/  (2010)  3,119/  Both/  _/  Urban | 35-74 | FBS ≥ 126 mg/dL | 65.5  (59.9, 70.8) | 59.6  (53.9, 65.1) | 28.3  (24.0, 34.4) | 6 |
|  | Lerner, 2013  ^226^ | Population based/  (2007 and 2008)  987/  Both/  47.2/  Both | >30 | FBS ≥ 126 mg/dL, or upon a self-report of a physician diagnosis | 69.7  (51.3, 84.4) | 24.2  (11.0, 43.0) | 3.0  (0.0, 15.8) | 5 |
|  | Segundo N Seclen,  ^227^ | Population-based/  (2010-2012)/  1,677/  Both/  -/  Urban | >25 | FBS ≥ 126 mg/dL | 60.2  (50.7, 69.1) | _ | _ | 6 |
| Panama | Donald P,  2013  ^228^ | Population based/  (2013)  3,590/  Both/  29.9/  Both | >18 | An individual who reported having a medical diagnosis of DM, FBS ≥ 126 mg/dL and/or HbA1c  ≥ 6.5% (≥ 48 mmol/mol) | 77.1  (72.2, 81.4) | 60.0  (54.6, 65.2) | 32.1  (27.1, 37.3) | 8 |
| Mexico | A. Basto-Abreu, 2021  ^229^ | Population based/  (2020)  1,175/  both/  33.3/  both | ≥20 | FBS ≥ 126 mg/dL  Control: HbA1C < 7% | 70.7 (68.0-73.3) |  | 39.0 (32.5-45.8) | 7 |
|  |  | Population-based/  (5897)/  2018/  both/  33.3/  both |  |  | 61.9 (60.6-63.1) | _ | 42.1 (38.4-45.9) |  |
|  | D. Aguilar 2021^230^ | Population based/  (1998-2004)/  9,9623/  both/  urban | 45-84 | HbA1C ≥ 6.5% or higher  Control: HbA1C < 7% | 76.0  (75.5-76.5) | 60.8  (60.2-61.4) | 15.8  (15.3-16.2) | 9 |
|  |  | Population based/  (2015-2019)  8,986/  both/  urban | 45-84 | HbA1C ≥ 6.5% or higher  Control: HbA1C < 7% | 89.0  (87.8-90.0) | 83.4 (82.3-84.9) | 36.8  (35.1-38.5) |  |
|  | S. Barquera, 2021  ^231^ | Population based/  2015/  1,307/  Both/  46.9/  Urban | 20-69 | FBS ≥ 126 mg/dL or HbA1c ≥ 6.5% | 70.5  (64.8, 75.7) | 58.6  (54.0, 63.1) | 17.5  (14.1-21.2) | 7 |
|  | Salvador Villalpando, 2010  ^232^ | Population-based/  (2006)/  6350/  Both/  _/  Both | >20 | FBS ≥ 126 mg/dL | 50.9  (47.5, 54.4) | _ | _ | 7 |
| Colombia | Vecino-Ortiz, 2012  ^233^ | Population-based/  (2007)/  12,496 | 18-69 | FBS ≥ 126 mg/dL | 70.4  (66.6, 74.0) | _ | _ | 8 |
| Kuwait | Alkandari,  2018,  ^234^ | Population-based/  (2014)/  2,561/  47.5/  Both | 36.4  18-69 | FBS ≥ 126 mg/dL or an HbA1c  ≥6.5% (48 mmol/mol). | 58.5  (54.0, 63.0) | 56.1  (51.6, 60.2) | _ | 6 |
| Qatar | A. Bener 2009  ^235^ | Population based/  (2008)  1,117/  both/  51.2/  urban | 20-59 | FBS ≥ 126 mg/dL or an OGTT ≥ 200 mg/dL | 64.5  (57.1-71.4) | _ | _ | 8 |
| Ireland | Leahy, 2015  ^236^ | Population-based/  (2009-2011)/  5,337/  Both/ | >50 | Self-reported doctors' diagnosis or taking diabetes medication | 84.2  (80.5, 87.4) | _ | _ | 6 |
| Brazil | E. S. M. dos Santos, 2020  ^237^ | Elderly based/  1,947/  (data of a cohort study)/  both/both | 62 | HbA1C ≥ 6.5% | 76.9  (72.9-80.5) | _ | _ | 7 |
|  | Maria Inês Schmidt, 2014  ^238^ | Population-based/  (2008-2010)/  15,102/  Both/  45.6/  Both | 52.1  >35 | FBS ≥ 126 mg/dL , 2-hour plasma glucose during the OGTT (2-HPP≥ 200 mg/dL ), or HbA1C  (≥6.5%; ≥47.5 mmol/mol) | 43.3  (41.4, 45.1) | _ | _ | 8 |
|  | Domingos A,  1992,  ^239^ | Population-based/  (1986-1988)/  21,847/  Both/  Urban | 30-69 | subjects without a history of diabetes who fasting capillary or RBS > 200 mg/dL at screening were  considered to have diabetes | 36.3  (34.3, 38.3) | _ | _ | 5 |
| Iraq | Mansour, 2014,  ^240^ | Population-based/  (2011-2012)/  5,445/  Both/  47.3/  Urban | 46.7±14.3  19-94 | FBS ≥ 126 mg/dL , HbA1c >6.5% (48 mmol/mol) were considered to indicate diabetes | 44.1  (41.1, 47.1) | _ | _ | 6 |
|  | B. Kasem 2014  ^241^ | Population based/  2011/  5,445/  both/  47.3/  both | 46.7 ± 14.3 19-94 | FBS ≥ 126 mg/dL   OGTT ≥ 200 mg/dL  HbA1C ≥ 6.5% | 44.2  (41.2-47.2) | ­_ | ­_ | 9 |
|  | A. A. Mansour 2008  ^242^ | Population-based/  2007  3,176/  both/  43.2/  both | 43.17 ± 16.37 ≥ 20 | FBS ≥ 126 mg/dL | 71.2  (64.9-76.9) | _ | _ | 8 |
| Palestine | A. Husseini 2001  ^243^ | Population-based/  1997/  492/  both/  38.6/  urban | 30-65 | OGTT ≥ 200 mg/dL | 78.0  (65.3-87.7) | 64.4  (50.9-76.4) |  | 6 |
|  | A. Husseini 2000  ^244^ | Population-based/  500/  both/  41.8/  rural | 30-65 | OGTT ≥ 200 mg/dL | 71.4  (56.7-83.4) | _ | _ | 5 |
| UAE | H. Saadi 2010  ^245^ | Population-based/  373/  both/  urban | ≥30 | FBS ≥ 126 mg/dL  OGTT ≥ 200 mg/dL previous diagnosis or oral antidiabetic treatment | 58.4  (48.2-68.1) | _ | _ | 5 |
|  | M. Malik 2005,  ^246^ | Population-based/  5,844/  both/  43/  both | ≥ 20 | Previous diagnosis or oral antidiabetic treatment  FBS ≥126 mg/dL | 59.4  (56.5-62.2) | _ | _ | 5 |
| Mauritania | Meiloud, 2013  ^247^ | Population-based/  1748/  Both/  48/  Both | >20 | FBS ≥126 mg/dL | 88.5  (85.5, 91.1) | _ | _ | 5 |
| Croatia | Metelko, 2008,  ^248^ | Population-based/  (1995-1997)  1,635/  Both/  50.2/  Both | 41.49 ±12.18  18-64 | FBS ≥126 mg/dL | 58.0  (47.7, 67.8) | _ | _ | 5 |
| New Zealand | Coppell, 2013  ^249^ | Population based/  (2008-2009)/  4721/  both/  both | ≥ 15 | Doctor diagnosed or self-report/ HbA1C ≥ 6.5% | 69.8 (64.5-74.7) | _ | _ | 7 |
|  | Gerhard Sundborn, 2007  ^250^ | Population-based/  (2002-2003)/  4,049/  Both/  48.0/  Both | 35-74 | FBS ≥126 mg/dL or 2-HPP ≥ 200 mg/dL | 82.1  (67.1, 76.7) | _ | _ | 6 |
| Australia | C. Glatthaar, 1985  ^251^ | Population-based/  8,301,358/  1981/  both/  49.3/  both | ≥ 25 | FBS ≥140 mg/dL | 72.8  (72.6-72.9) | _ | _ | 8 |
| Japan | M. Goto 2015  ^252^ | Population-based /  20496/  both/  40.6/  both | ≥ 20 | HbA1C ≥ 6.5% | _ | 61.9 (59.7-64.0) | _ | 8 |
|  | Akira sekikawa, 1993  ^253^ | Population-based/  (1990)/  916/  42.5  Both/  45.8/  Urban | >45 | For international comparisons, the 1980 WHO criteria were used to determine the results of OGTTs | 47.7  (38.0, 57.5) | - | - | 5 |
| Korea | Sunjoo Boo, 2018  ^254^ | Population based/  (2013)/  136,655/  Both/  51.6/  Both | 60-64 | A self-reported history of diabetes or an FBS ≥ 126mg/dL | 61.81  (61.0, 62.6) | _ | 26.2  (25.3, 27.0) | 8 |
|  | Hyun Ja Kim, 2014  ^255^ | Population based (2005)/  4,599/  Both/  42.8/  Both | ≥30 | FBS ≥ 126 mg/dL, a previous diagnosis of diabetes by  physician, or current use of anti-diabetic agents or insulin | 68.2  (63.5, 72.6) | 49.9  (45.0, 54.8) | 22.1  (18.2, 26.4) | 7 |
|  |  | Population based (2007)/  13,931/  Both/  42.5/  Both |  |  | 72.4  (70.0, 74.8) | 50.0  (46.9, 52.2) | 28.1  (25.8, 30.6) |  |
|  |  | Population based (2010)/  14,655/  Both/  42.8/  Both |  |  | 73.1  (70.7, 75.3) | 63.2  (60.6, 65.7) | 28.9  (26.6, 31.3) |  |
|  | Eun-Hee Cho, 2017  ^256^ | Population based/  83,577 (2005)/  Both/  52.0  Both | 20-29 | FBS ≥126 mg/dL and/or HbA1c levels of 6.5% or higher (≥ 47 mmol/mol) | 84.9  (84.6, 85.3) | 19.7  (19.3, 20.1) | _ | 8 |
|  |  | Population based/  83,577 (2007-2009)/  Both/  52.3  Both |  |  | 63.9  (36.6, 64.3) | 11.9  (11.7, 12.2) | _ |  |
|  |  | Population based/  83,577 (2010-2012)/  Both/  52.5  Both |  |  | 52.5  (52.1, 52.9) | 6.2  (6.0, 6.4) | _ |  |
|  |  | Population based/  83,577 (2013-2014)/  Both/  52.7  Both |  |  | 18.0  (17.7, 18.3) | _ | _ |  |
|  | Lee, 2018  ^257^ | Population-based/  (2005)/  4,603/  Both/  42.7/  Both | >30 | FBS ≥126 mg/dL | 71.8  (67.3, 75.9) | _ | _ | 6 |
|  |  | Population-based/  (2007-2009)/  13,931/  Both/  42.4/  Both |  |  | 76.6  (74.4, 78.7) | _ | _ |  |
|  |  | Population-based/  (2010-2012)/  14,768/  Both/  42.8/  Both |  |  | 77.7  (75.6, 79.6) | _ | _ |  |
|  |  | Population-based/  (2011-2013)/  12,855/  Both/  42.6/  Both |  |  | 69.9  (67.3, 72.0) | _ | _ |  |
|  | S. M. Kim 2006  ^258^ | Population based/  2001/  5,844/  both/  43/  both | > 20 | FBS ≥ 126 mg/dL | 57.9  (53.5-62.2) | _ | _ | 9 |
| OGTT: oral glucose tolerance test, HbA1C: glycosylated hemoglobin, FBS: fasting blood sugar RBS: random blood sugur; 2-HPP: 2-hour post-prandial blood glucose | | | | | | | | |
